# Agonist-selective activation of individual G-proteins by muscarinic receptors

**DOI:** 10.1038/s41598-024-60259-4

**Published:** 2024-04-26

**Authors:** Dominik Nelic, Nikolai Chetverikov, Martina Hochmalová, Christina Diaz, Vladimír Doležal, John Boulos, Jan Jakubík, Kirill Martemyanov, Alena Janoušková-Randáková

**Affiliations:** 1 Department of Neurochemistry, Institute of Physiology, Czech Academy of Sciences, Prague, Czech Republic; 2 Department of Physical Sciences, Barry University, Miami Shores, Miami, FL USA; 3 Department of Neuroscience, UF Scripps Biomedical Research, University of Florida, Jupiter, FL 33458 USA

**Keywords:** Biochemistry, Biological techniques, Cell biology, Chemical biology, Drug discovery, Molecular biology, Neuroscience

## Abstract

Selective activation of individual subtypes of muscarinic receptors is a promising way to safely alleviate a wide range of pathological conditions in the central nervous system and the periphery as well. The flexible G-protein interface of muscarinic receptors allows them to interact with several G-proteins with various efficacy, potency, and kinetics. Agonists biased to the particular G-protein mediated pathway may result in selectivity among muscarinic subtypes and, due to the non-uniform expression of individual G-protein alpha subunits, possibly achieve tissue specificity. Here, we demonstrate that novel tetrahydropyridine-based agonists exert specific signalling profiles in coupling with individual G-protein α subunits. These signalling profiles profoundly differ from the reference agonist carbachol. Moreover, coupling with individual Gα induced by these novel agonists varies among subtypes of muscarinic receptors which may lead to subtype selectivity. Thus, the novel tetrahydropyridine-based agonist can contribute to the elucidation of the mechanism of pathway-specific activation of muscarinic receptors and serve as a starting point for the development of desired selective muscarinic agonists.

## Introduction

Muscarinic acetylcholine receptors are G-protein coupled receptors (GPCRs) widely expressed both in the central nervous system (CNS) and the periphery. Depending on the muscarinic receptor subtype (M1–M5) and location, they mediate a variety of physiological functions^1^. Individual subtypes differ in signalling profiles. Subtypes M1, M3, and M5 preferentially activate the Gq/11 class of G-proteins, while M2 and M4 preferentially activate inhibitory Gi/o G-proteins^1^. The flexible G-protein interface of muscarinic receptors allows them to interact with several types of G-proteins with various efficacy, potency, and kinetics^2^. Moreover, muscarinic receptors can also couple to G-proteins from other classes^3^.

Disruption of muscarinic signalling often contributes to pathologies in the CNS and periphery^4^ making muscarinic agonists potential tools for the treatment of a variety of pathologies including Alzheimer’s disease (M1)^5^ and neuropathic pain (M2)^6–8^. Importantly, selective modulation of individual subtypes of muscarinic receptors is necessary to avoid undesired side effects. Many muscarinic agonists targeting diseases of the CNS have failed in clinical trials mainly due to gastrointestinal and cardiovascular adverse effects attributed to the activation of peripheral M2 and M3 receptors^9,10^. High homology of the orthosteric binding sites across subtypes of muscarinic receptors makes the development of agonists with selectivity for a particular receptor subtype extremely challenging^11–13^.

While many G-protein types are ubiquitously expressed, some are preferentially found in specific tissues^14^. For example, Gi1, Gi2, and Gi3 types of inhibitory G-proteins are widely expressed in the whole body, while type GoA is predominantly found in the brain^15–17^. Studies with mice lacking the Gαo have reported several neurological deficits, including tremors, seizures, repetitive turning behaviour, abnormal exploratory behaviour or poor motor coordination and exhibit hyperalgesia when subjected to a hot plate test^18^. Highly specific tissue distribution was also observed for G15 and G14 belonging to the PLC-activating Gq/11 class of G-proteins. While G11 and Gq are widely distributed, G14 is more expressed in the thyroid^19^, spleen, lung, kidney, and testis^20,21^. G15 is primarily restricted to hematopoietic lineages^20^ and epithelial cells, which are characterized by a high rate of cell turnover^22,23^.

Upon receptor activation, transmembrane helices of a given GPCR: TM3, TM5, TM6, and TM7 tilt and rotate creating a cavity at the intracellular side of the receptor into which the G-protein inserts the C-terminus of the α-subunit. Subsequently, G-protein becomes active and triggers a particular signalling pathway^24^. Receptors select G proteins with some specificity for a given G-protein type being constrained by the specific geometry of the receptor G-protein interface^25^. Structurally different agonists can stabilize different active conformations of the receptor with different geometry of the G-protein interface thereby leading to selective engagement of downstream signalling pathways termed *signalling bias*^26,27^. It has been demonstrated that structurally different agonists display specific activation patterns of individual G-proteins at the particular muscarinic subtype^2,28–30^. Thus, selective targeting of a subset of a particular G-protein mediated signalling pathways by biased agonists can be used to achieve selectivity among individual subtypes of muscarinic receptors and may lead to tissue-specific activation due to preferential expression of specific G-proteins. Therefore such agonists may be of interest for the development of more selective therapeutic agents with fewer side effects^31^.

An in-depth understanding of the biased signalling of agonists is an indispensable step for the determination of structure**-**activity relationships of agonists for a particular signalling pathway and for developing pathway-specific selective drugs with fewer side effects The binding of an agonist to so-called ‘hot spot’ residues that dictate activation of a specific signalling pathway^32^ is thought to be key in determining ligand-mediated signalling bias. According to this model, an agonist substantially smaller than the orthosteric pocket has a higher probability of binding to fewer functional hot spots and exerting signalling bias.

Recently, we have identified a novel orthosteric partial muscarinic agonist (1-methyl-1-[(thiophen-2-yl)methyl]-1,2,3,6-tetra hydropyridin-1-ium) JR-6. Compared to a reference agonist carbachol (CBC), JR-6 showed greater influence on cAMP inhibition by M2 and M4 receptors relative to the effects of M1 and M3 receptors on the accumulation of inositol phosphates in CHO cells. This bias was also observed in the endogenous tissues with JR-6 diminishing cAMP levels in the striatum which predominantly expresses the M4 receptor and heart atrium which predominantly expresses M2 to a greater extent than it regulated accumulation of inositol phosphates in rat cortex and salivary glands predominantly expressing M1 and M3 receptors, respectively^33^.

In this study, we examined the concept of biased signalling at muscarinic receptor subtypes by taking advantage of the small pharmacophore of JR-6 and modifying it to develop a set of structurally diverse muscarinic agonists. We paired the pharmacological studies with real-time measurements of G protein selectivity of muscarinic receptor types at the level of the receptor-G protein interaction to avoid interference from the downstream cross-talk. Our studies document substantial G protein biases set by selective agonists at different types of muscarinic receptors.

## Results

### Rational design of M2-biased compounds

**Figure 1:**
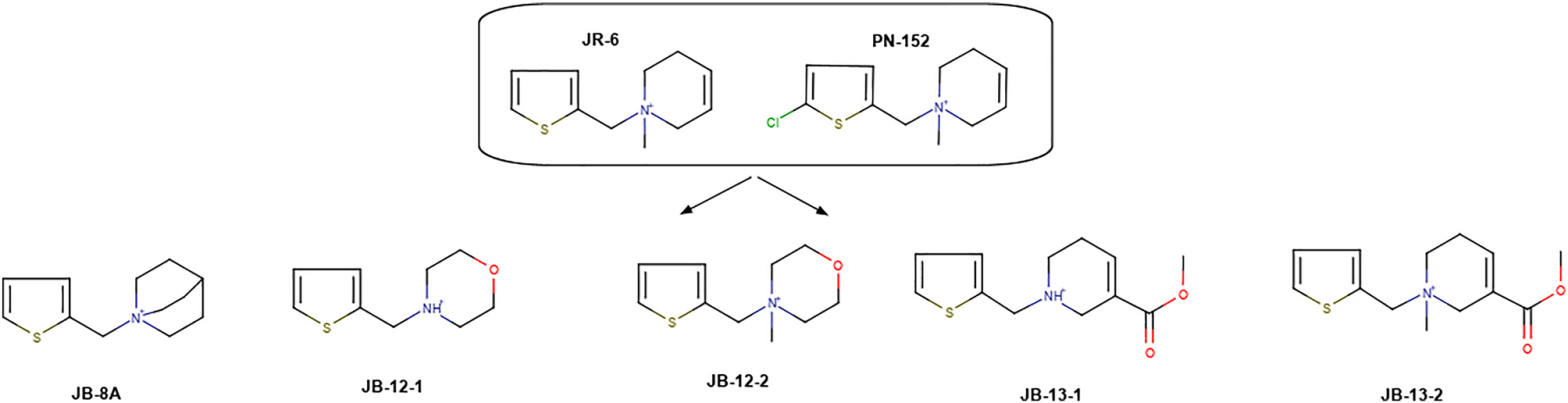
Structures of novel agonists. Top, published compounds JR-6 (hit compound 7A) and its chlorine derivate PN-152 (7D)^33^. Left, novel analogues with the THP ring were replaced by quinuclidine (JB-8A) or morpholine (JB-12-1 and JB-12-2). Right, novel analogues with methyl ester were added to position 5 of the THP ring (JB-13-1 and JB-13-2).

Compound JR-6 (1-methyl-1-[(thiophen-2-yl)methyl]-1,2,3,6-tetra hydropyridin-1-ium) was previously described as Gi/o-biased and M2 functionally selective partial agonist^33^. In contrast, its chlorinated analogue PN-152 (1-[(5-chlorothiophen-2-yl)methyl]-1-methyl-1,2,3,6-tetrahydropyridin-1-ium) was a partial agonist at all subtypes of muscarinic receptors. Chlorination of the thiophene ring (PN-152) caused the loss of M2-biased properties^33^. The principal difference between parental compounds (JR-6 and PN-152) and canonical muscarinic agonists is the π-π stacking interaction between the thiophene ring and W400^34^. Therefore, we focused on modifications of the tetrahydropyridine (THP) ring to preserve the M2 bias of the parental compound JR-6 and improve its binding affinity and potency. We designed and synthesized a set of JR-6 analogues with modifications at the THP ring (Fig. 1). First, we introduced quinuclidine (1-azabicyclo[2.2.2]oct-3-yl), a bicyclic moiety responsible for the high affinity of 3-Quinuclidinyl benzilate (JB-8A). Quinuclidine is also part of the relatively potent agonist AF102B^35^. The second modification was the introduction of morpholine ring (JB-12-1 and JB-12-2) that is present in some potent muscarinic agonists^36^. The third modification of the THP ring was the addition of methyl ester group as found in the structure of arecoline (JB-13-1 and JB-13-2). Except for JB-8A and JB-12 compounds, ligands possess chiral nitrogen. All chiral compounds were synthesized and tested as racemates. Synthesis is described in the supplementary information (SI) Fig. S1A–D.

### Impact of changes in compound scaffold structure on the binding affinity to individual subtypes of muscarinic receptors

The affinity of novel agonists was determined by competition with ^3^H-N-methylscopolamine (NMS) binding to membranes from CHO cells stably expressing individual subtypes of muscarinic receptors. The equilibrium dissociation constant (K_D_) for [^3^H]NMS was 0.1 nM for M1, M3, M4, M5 and 0.4 nM for M2 receptors, respectively. The expression level was from 1.4 ± 0.03 (M5) to 5.59 ± 0.12 (M1) pmol/mg of membrane protein. The affinity of JR-6 was around 10 µM and three to ten times higher for PN-152 (Table 1). Introducing the quinuclidinyl group (JB-8A) increased the binding affinity at the M2 receptors, and slightly decreased it at the M3. The introduction of morpholine (JB-12) decreased the binding affinity to all subtypes as well as the substitution by methyl ester (JB-13). N-methylated analogues had higher affinity than non-methylated counterparts. Designed changes in the THP ring led to negative changes in binding affinity at all subtypes, except JB-8A at M2.

**Table 1:** Binding affinities of novel compounds with modification of THP moiety.

Subtype	Binding affinity						
	JB-8A	JB-12-1	JB-12-2	JB-13-1	JB-13-2	JR-6	PN-152
M1	5.16 ± 0.20	< 3^a^	4.07 ± 0.19^a^	3.94 ± 0.05^a^	4.37 ± 0.09^a^	4.95 ± 0.07	5.86 ± 0.08
M2	5.49 ± 0.05^b^	3.52 ± 0.2^a^	4.76 ± 0.06	4.63 ± 0.06^a^	5.02 ± 0.30	5.10 ± 0.10	6.16 ± 0.06
M3	4.83 ± 0.06^a^	< 3^a^	4.03 ± 0.04^a^	3.45 ± 0.16^a^	3.90 ± 0.14^a^	5.10 ± 0.10	5.47 ± 0.02
M4	5.34 ± 0.08	< 3^a^	4.77 ± 0.08^a^	4.11 ± 0.09^a^	4.64 ± 0.20^a^	5.10 ± 0.10	5.51 ± 0.06
M5	5.03 ± 0.12	< 3^a^	3.95 ± 0.12^a^	3.33 ± 0.07^a^	4.01 ± 0.16^a^	5.00 ± 0.20	5.77 ± 0.05

Inhibition constants of indicated compounds (Ki) to individual subtypes of muscarinic receptors were calculated according to Eq. (2) from IC_50_ values that were obtained by fitting Eq. (1) to data from competition experiments with [^3^H]NMS. Values are expressed as a negative logarithm of Ki. Values are mean ± SD from three independent experiments performed in quadruplicates.

Significantly different (P < 0.05) according to one-way ANOVA followed by Dunnett’s multiple comparison test. For reference, pKi values of compounds JR6 and PN-152 by Randáková et al.^33^ are included.

^a^Lower then pKi of JR-6 at given muscarinic subtype.

^b^Higher then pKi of JR-6 at given subtype.

### THP-based muscarinic agonists differentially impact GTPγ[^35^S]binding to Gα i/o subtypes

To obtain insight into the impact of THP-based agonists on muscarinic signalling, we began by analyzing their ability to induce activation of individual members of Gi/o class (GαoA, GαoB, Gαi1, Gαi2 and Gα3). We used Sf9 cell membranes containing individual combinations of the M2 receptor (expression level 4.41 ± 1.12 pmol/mg of membrane protein) and given Gαi/o subunit^37^ to examine the binding of radiolabeled GTPγ[^35^S] to Gα subunits in response to agonist application. The endogenous Gβγ subunits were sufficient for the GTPγ[^35^S] assay. This assay enables the determination of agonist potency and efficacy without amplification or other modulation that may occur during downstream signalling of the receptor.

**Figure 2:**
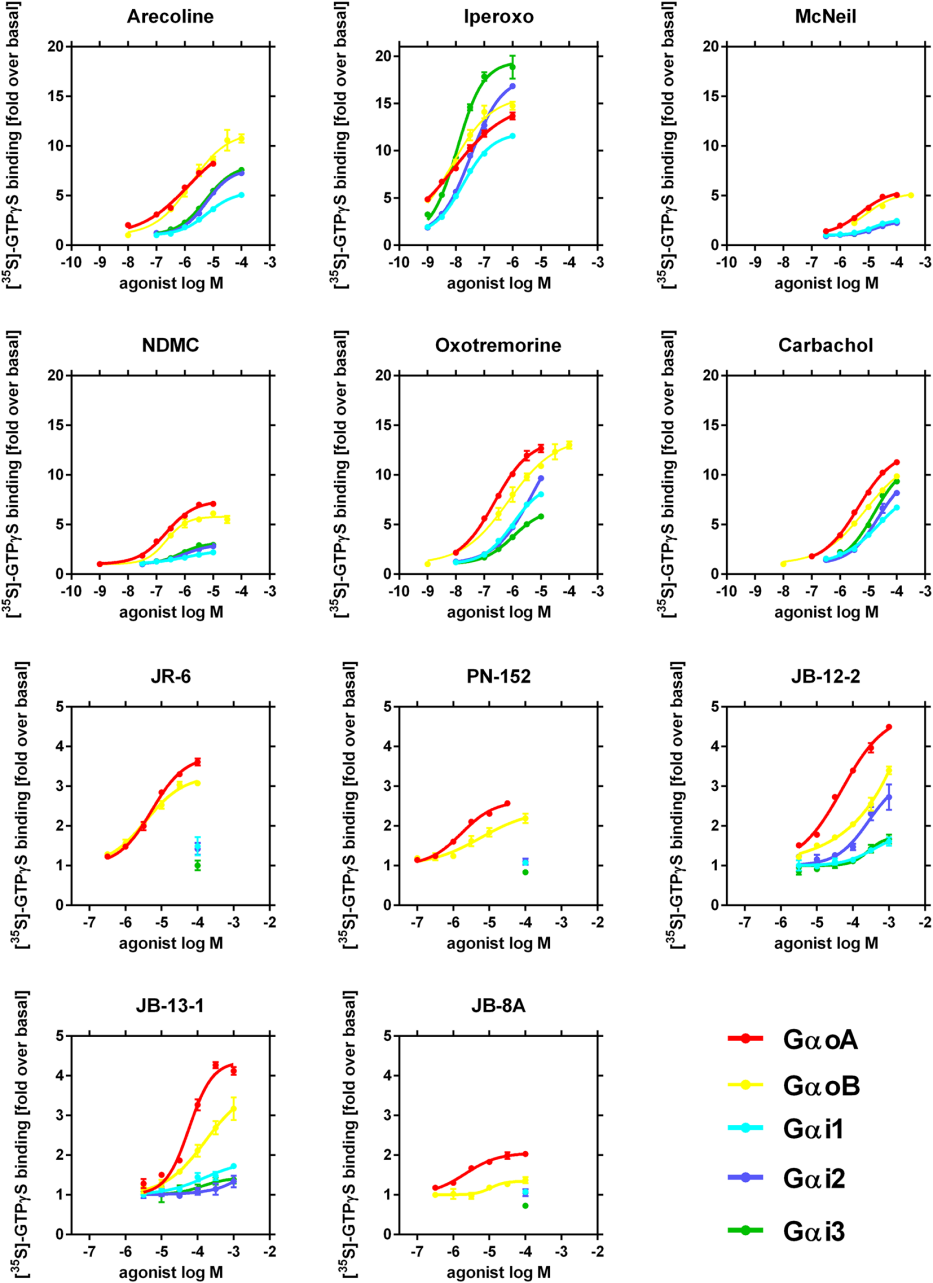
Agonist-induced activation of individual types of Gαi/o on M2 receptor. Y-axis, functional responses measured as agonist-induced GTPγ[^35^S]binding to membranes from Sf9 cells expressing a particular combination of M2 receptor with a given Gαi/o (indicated in the legend) are expressed as a fold increase over the basal level (in the absence of agonist). X-axis, the molar concentration of agonists is expressed as a logarithm. Data are means ± S.E.M from representative experiments performed in quadruplicates.

Representative experiments are shown in Fig. 2 and parameters of agonist potencies and efficacies to stimulate GTPγ[^35^S]binding to a given α-subunit of G-protein EC_50_ and E’_MAX_ are summarized in SI (Table S1) together with the calculated parameters of the operational efficacy τ and the equilibrium dissociation constant K_A_.

All evaluation is done according to standard procedures for evaluation of biased signalling using CBC as a reference agonist (Kenakin^38^, Kenakin et al.^39^, Grifin et al.^40^, see “Data analysis”).

**Figure 3:**
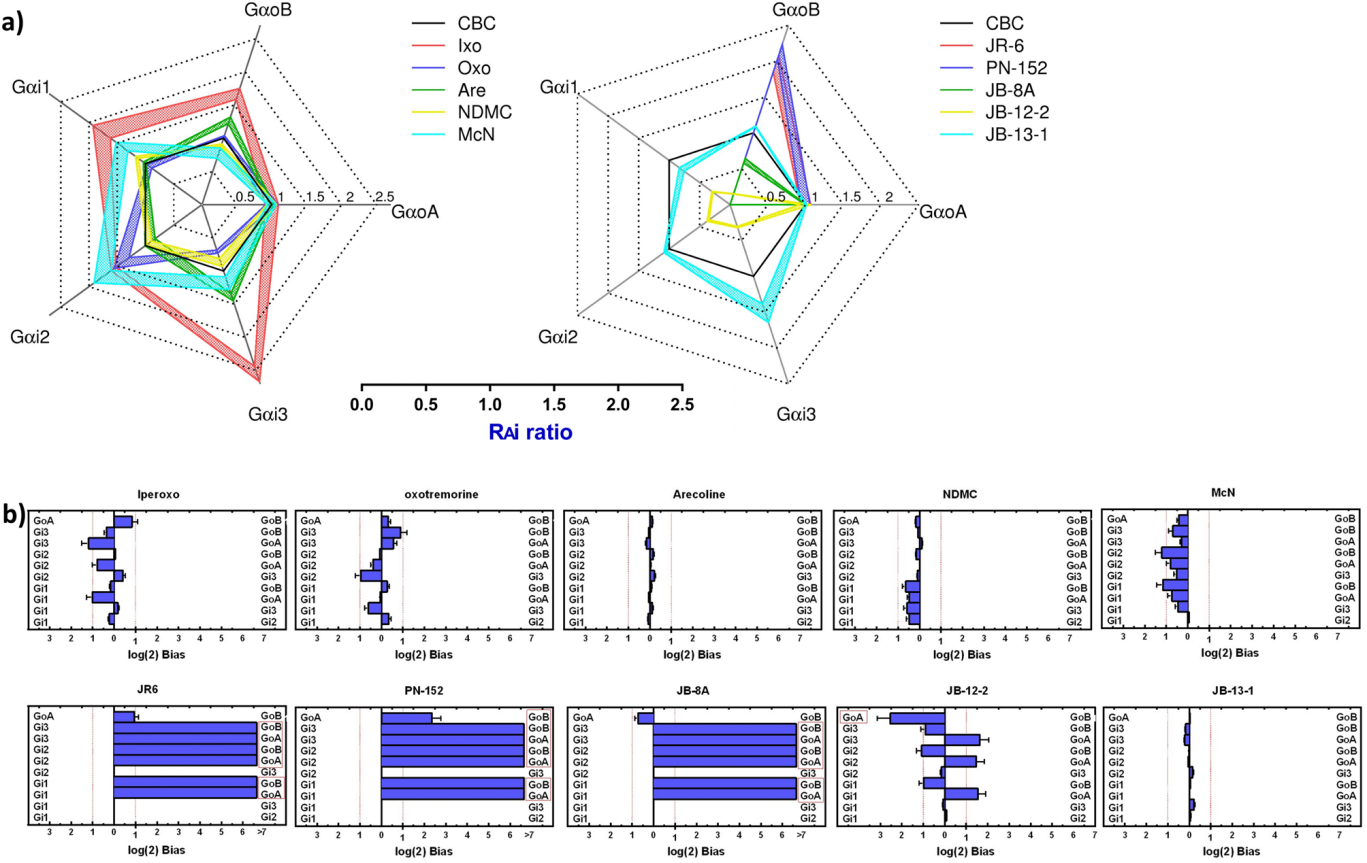
Signaling bias among individual Gi/o proteins activated by M2. (**a**) spider plots show relative intrinsic activity RAi at individual Gαi/o types for agonists (indicated in legend). Intrinsic activities of individual agonists relative to reference agonist carbachol (RAi) calculated according to Eq. (7) from the measurement of GTPγ[^35^S]binding (Table S2) are plotted as ratios to RAi at GαoA for a given agonist. (Values of reference agonist carbachol are equal to 1). The thickness of the line represents confidence intervals of calculated RAi values. (**b**) bias plot among individual types of Gi/o G-protein. Values are expressed as log(2) of the bias factor, where the bias factor is calculated according to Eq. (8). Values > 1 (more than two-fold preference) were taken as relevant and displayed in a red frame. Values > 7 mean no response at the Gα subunit in the left column. Data of RAi and bias factors are summarized in SI (Tables S2 and S3, respectively).

To quantify agonist bias in the activation of individual Gαi/o relative to reference agonist, intrinsic activities relative to carbachol (RA_i_) were calculated according to Grifin et al.^40^ from the E’_MAX_ and EC_50_ values according to Eq. (7). Values of RA_i_ for individual agonists at a given Gαi/o are summarized in SI (Table S2) and plotted in Fig. 3A. Further, we calculated the bias factor between two particular Gαi/o types according to Kenakin et al.^39^, based on the ratio of operational efficacy τ and equilibrium dissociation constant K_A_ (SI, Table S1). Bias factors are plotted in Fig. 3B and summarized in SI (Table S3). Taking into account 5% relative SD in technical replicates in the GTPγS binding assay, the detection threshold of the assay is about a 10% increase in the basal value. JR-6 produces a signal more than 20-fold the detection limit at GoA and no signal at Gi1. The signal of CBC at Gi1 is 62% of GoA. Therefore for JR-6, up to 14-fold bias in the GoA vs. Gi1 pathway can be quantified. Similarly, up to 17-fold bias for GoA vs. Gi2, and 13-fold bias for GoA vs. Gi3 can be quantified for JR-6. Ligand exerting profound activity at one pathway and no activity at another is deemed to possess absolute signalling selectivity.

Reference agonists CBC activated all Gi/o types (Fig. 2) with similar operational efficacy τ (SI, Table S1). Unlike CBC, JR-6 as well as its analogues PN-152 and JB-8A activated GαoA and GαoB only (Fig. 2). Operation efficacy of these ligands at GαoA and GαoB was lower than the efficacy of CBC (SI, Table S1) (approx 10× for JR-6, 15× for PN-152 and 20–30× for JB8A at GαoA and GαoB, respectively). Relative to CBC, these compounds displayed absolute selectivity to Gαo with a preference for GαoB (Fig. 3A). Compounds JB-12-2 and JB-13-1 activated all Gi/o Gα (Fig. 2) with the highest operational efficacy at GαoA (SI, Table S1). Relatively to CBC, JB-12-2 was biased to GαoA and JB-13-1 to Gαi3 (Fig. 3A).

Our results have shown that novel THP-based agonists display profoundly different profiles than CBC and the profiles vary also among these ligands (Fig. 3A, B). To determine whether this difference is a property of THP-based muscarinic agonists or if it is common among structurally different agonists, we analysed the signalling profile of five structurally diverse muscarinic agonists with distinct potencies and efficacies and calculated signalling bias relative to carbachol. For this analysis, we have chosen superagonist iperoxo, classical muscarinic agonists oxotremorine and arecoline, and atypical agonists NDMC and Mc-Neil A343. All these agonists activated all Gi/o Gα (Fig. 2) with various operational efficacies (SI, Table S1). Unlike some THP-based agonists with absolute selectivity towards Gαo (JR-6, PN-152 and JB-8A) or more than 5-fold bias to GoA vs GoB (JB-12-2), signalling profiles of control agonists display only small variations (Fig. 3A, B). According to RAi, the superagonist iperoxo displayed slight bias to Gαi3 (2.5 times more than GαoA), oxotremorine to Gαi2 (2× vs Gαi3) and McN to Gαi2 (2.3× vs GαoB) (Fig. 3A, SI, Table S2).

The functional response of reference agonist CBC (Fig. 2) may indicate putative system bias towards GαoA. It should be noted that GαoA is not the most efficiently coupled α-subunit. Rather the coupling efficiency is ligand-dependent as significant differences were found. Iperoxo has more than twice “better” coupling to Gαi3 than to GαoA. Taking CBC as a reference agonist, M2 coupling to GαoB was almost as good as to GαoA. Importantly, the activity of JR-6 and PN-152 was greater at GαoB than at GαoA, being biased to GαoB, and JB-13-1 is biased to Gαi3. Among THP-compounds, only JB-8A and JB-12-2 are biased to GαoA. This clearly demonstrates the ligand bias of the tested compounds.

### Real-time monitoring of G-protein activation with BRET confirms biased action of ligands at muscarinic receptors

**Figure 4:**
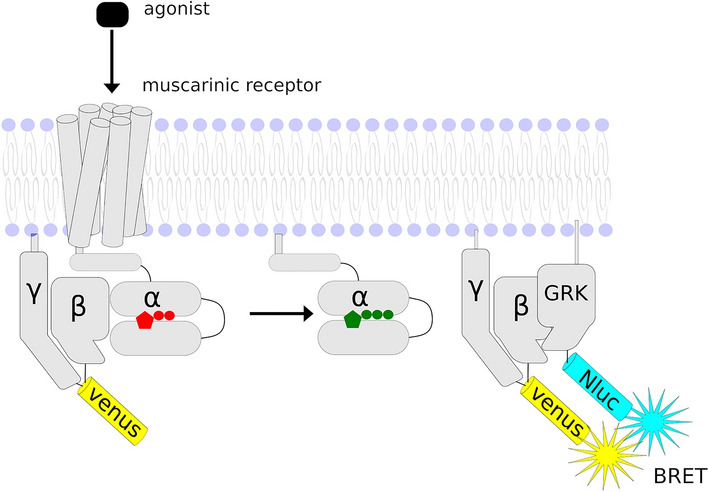
Schematic representation of the BRET assay for real-time optical imaging of G protein activity. Agonist-induced activation of a GPCR leads to the dissociation of Gα-GTP and Venus-Gβγ subunits. The released Venus-Gβγ then interacts with the Gβγ effector mimetic masGRK3ct-Nluc to produce the BRET signal.

We next extended our observations using a high-resolution BRET-based assay to study the activation of various G protein types by muscarinic receptors in real-time. This assay provides a quantitative readout of G-protein activation in living cells using a bystander strategy that monitors Gβγ release which allows us to assess the behavior of unmodified full-length Gα subunits and accurately compare their activation by GPCRs in parallel^41^ (Fig. 4). With this approach, we determined both the kinetics and extent of activation of individual G-proteins by muscarinic receptors in response to the saturating concentration of novel THP-based agonists JR-6, PN-152, JB8A, JB-12-2 and JB-13-1.

First, we focused on examining the coupling of the M2 receptor with individual members of Gi/o class: GαoA, GαoB, Gαi1, Gαi2, Gαi3 and Gαz as well as promiscuous Gα15 upon activation by JR-6 and its analogues. The expression level of the M2 receptor in this system was 0.28 ± 0.03 pmol/mg of membrane protein. Further, we analysed the coupling of muscarinic M1 and M3 receptors with members of the Gq/11 class: Gαq, Gα11, Gα14 and Gα15. The expression level of the M1 and M3 receptors in this system was 2.12 ± 0.04 and 1.63 ± 0.05 pmol/mg of membrane protein, respectively. This was accomplished by quantifying both maximal BRET response amplitudes and G-protein activation rates across individual G-proteins upon stimulation of receptors with a saturating concentration of given agonists. In all experiments, CBC, at the saturating concentration of 100 μM, was used as a reference agonist.

Application of agonists to cells lacking exogenous Gα subunits did not produce the BRET signal, indicating that the signal is specific to heterologously expressed Gα subunits (SI Fig. S3). We further ensured that responses induced by studied ligands are within the dynamic range of the assay, by showing that higher efficacy agonist acetylcholine can generate a response with amplitude higher than that of analyzed compounds (SI Fig. S3). These controls indicate that our system can distinguish relative differences in the coupling of receptors to individual G proteins.

**Figure 5:**
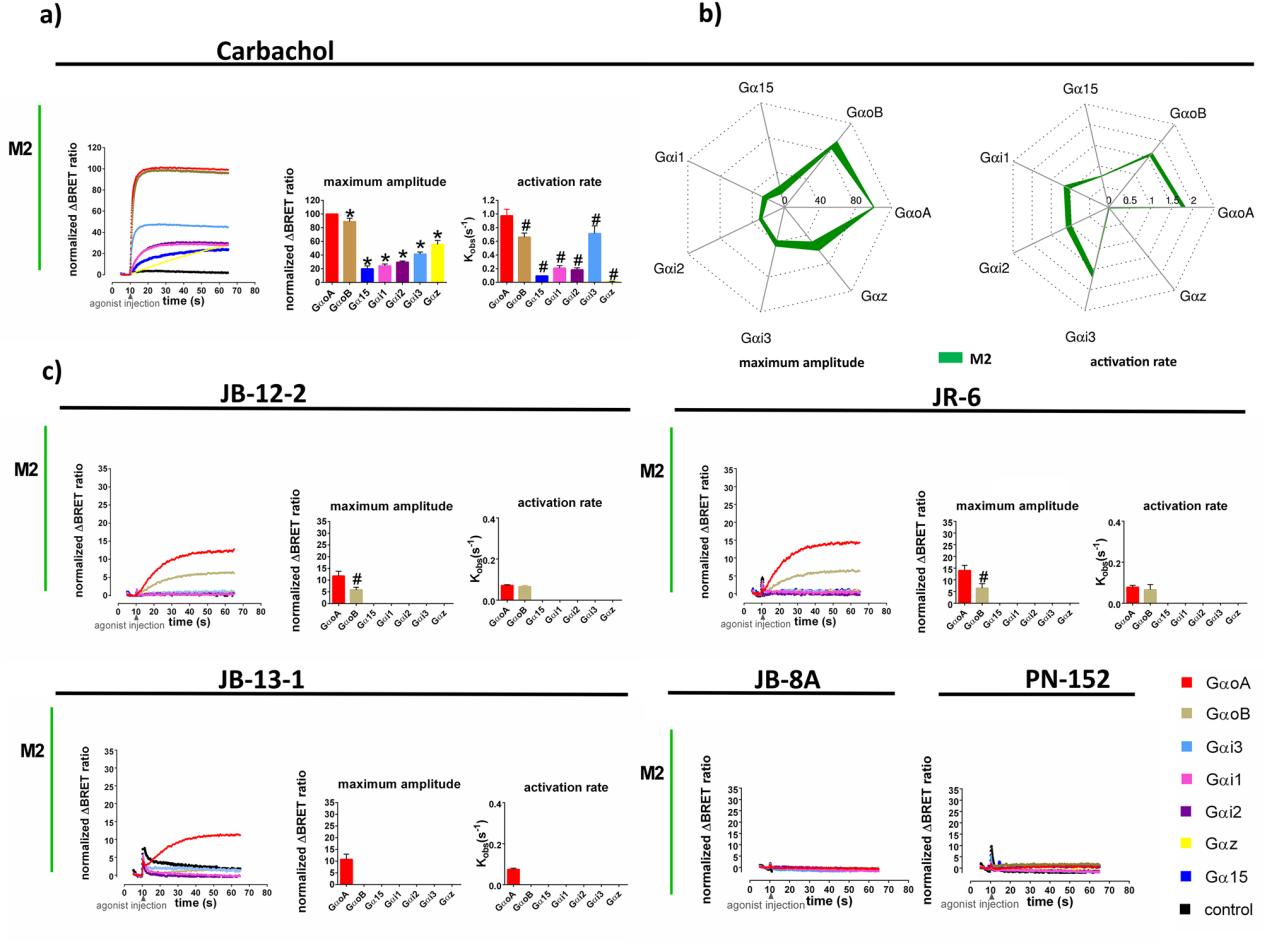
Agonist-induced activation of individual Gα subunits via the M2 receptor. Time-courses of the representative experiments of agonists-induced real-time monitoring of activation individual Gα subunits (indicated in the legend) induced by reference agonist CBC (100 μM) (**a**) THP-based compounds JB-13-1, JB-12-2 (1 mM), JR-6, JB-8A and PN-152 (100 μM) (**c**) are plotted in the x, y graphs (X-axis, time in seconds; Y-axis, amplitudes of Gα activation expressed as ΔBRET ratio normalized to % of the maximal response of the reference agonist CBC at GαoA). Traces are running averages over nine consecutive determinations. Bar plots show means ± SD of observed maximal amplitudes and activation rate constants Kobs, expressed in s-1, obtained by fitting Eq. (9) to the data from three independent experiments performed in triplicates. *P < 0.05 significantly different from the maximal response at GoαA (set to 100) induced by CBC according to one-sample *t*-test; #P < 0.05 significantly different from the response of given agonist at GoA, according to one-way ANOVA followed by Dunnet’s multiple comparison test or *t*-test as appropriate. Data of maximum amplitudes and activation rate constants of reference agonist CBC are also expressed in the polar plot (**b**), where amplitudes are expressed as % of maximal response at GαoA and activation rate constants as logarithms of the observed rate per minute. Line thickness represents the SD of three independent experiments performed in triplicates. Data are summarized in SI Table S4.

In this system, reference agonist CBC activated all tested Gαi/o types and promiscuous Gα15 via the M2 receptor with various maximal magnitudes and kinetics (Fig. 5A, B; SI Table S4). The magnitude of responses decreased in order GαoA > GαoB >  > Gαz > Gαi3 > Gi2 ~ Gαi1 ~ Gα15 and activation kinetics in order GαoA > Gαi3 ~ GαoB > Gαi1 ~ Gi2 ~ Gα15 ~ Gαz. In comparison with CBC, THP-based ligands produced very small or no activation of Gαi/o and Gα15 (Fig. 5C; SI Table S4). The signal kinetics was also much slower than in the case of carbachol (Fig. 5; SI Table S4). We detected a weak activation of GoA and GoB by JB-12-2 and JR6 reaching approximately 12% and 6% of CBC magnitude, respectively (Fig. 5C, SI Table S4). Both Go Gα were activated with similar kinetics (Fig. 5C; SI Table S4). In this assay, agonist JB-13-1 activated only GαoA at approximately 10% of CBC magnitude and the same kinetics as JR6 (Fig. 5C; SI Table S4). In comparison with GTPγ[^35^S]binding assay, we did not detect PN-152 and JB-8A induced activation of tested Gαi/o via the M2 receptor (Fig. 5C). Immense differences in CBC-induced maximal amplitude on GoαA, B and the rest of Gi Gα (e.g. Gi1 ~ 25% GoA) (Fig. 5A; SI Table S4) complicate the analysis of the coupling profile THP-based agonists exerting low efficacies. Analysis of the activation of Gαi and Gα15 types by weak agonists could be limited by assay sensitivity.

**Figure 6:**
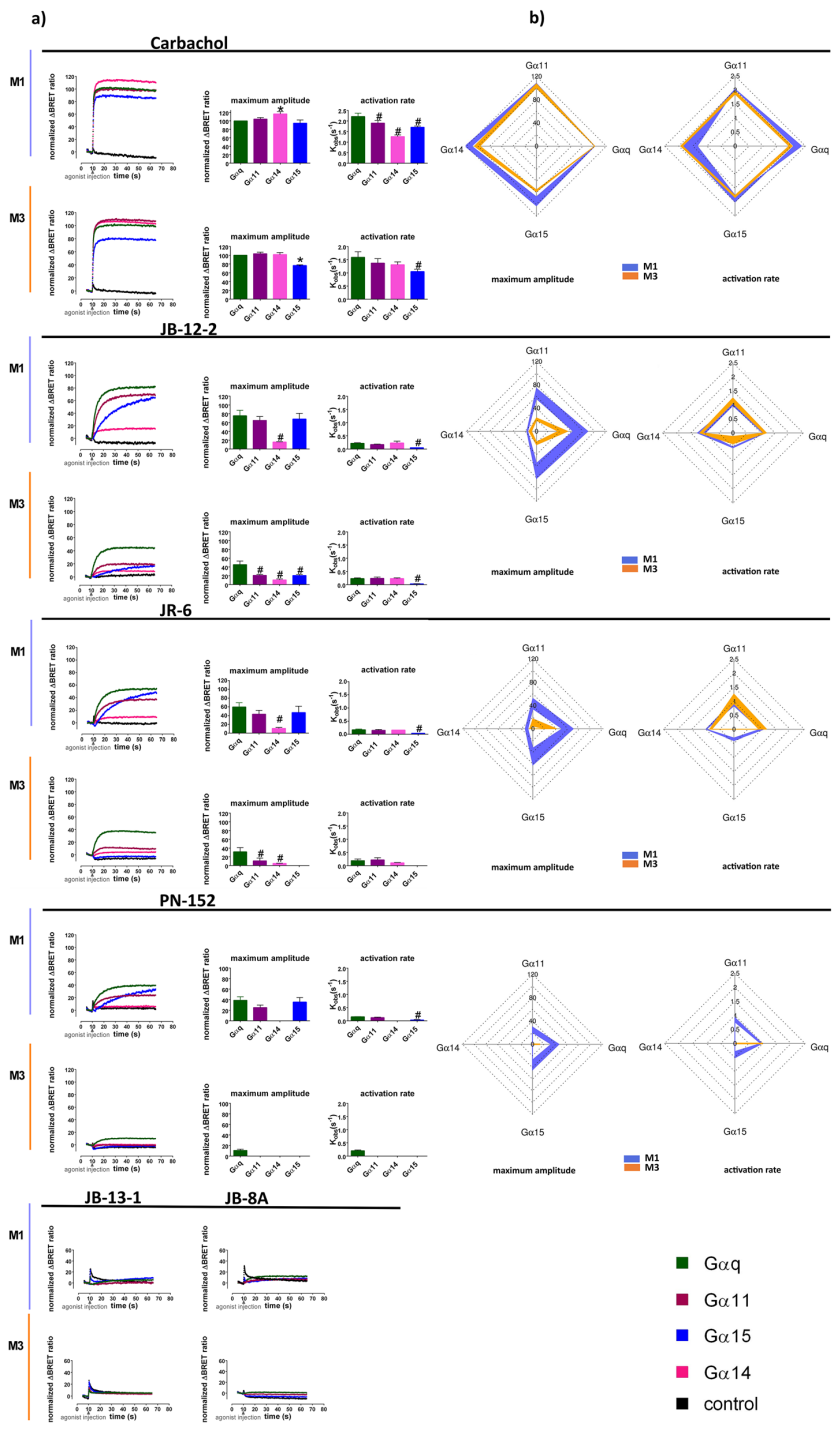
Comparison of agonist-induced activation of individual Gq/11 α subunits via the M1 and M3 receptors. Time-courses of the representative experiments of agonists-induced real-time monitoring of activation individual Gα subunits (indicated in the legend) induced by reference agonist CBC (100 μM) and THP-based compounds, JB-12-2, JB-13-1 (1 mM), JR6, PN-152 and JB-8A (100 μM) are plotted in the x, y graphs (X-axis, time in seconds; Y-axis, amplitudes of Gα activation expressed as ΔBRET ratio normalized to % of the maximal response of the reference agonist CBC at Gαq). Traces are running averages over nine consecutive determinations [(**A**) on the left]. Bar plots [(**A**) on the right] show means ± SD of observed maximal amplitudes and activation rate constants Kobs, expressed in s-1, obtained by fitting Eq. (9) to the data from at least three independent experiments performed in triplicates. *P < 0.05 significantly different from the maximal response at Gαq (set to 100) induced by CBC according to one-sample *t*-test; #P < 0.05 significantly different from the response of given agonist at Gq, according to one-way ANOVA followed by Dunnet’s multiple comparison test or *t*-test as appropriate. Data of maximum amplitudes and activation rate constants of individual agonists are also expressed in the polar plot (**B**), where amplitudes are expressed as % of maximal response at Gαq and activation rate constants as logarithms of the observed rate per minute. Line thickness represents the SD of at least three independent experiments performed in triplicates. Data are summarized in SI Table S5.

Although M1 and M3 receptors canonically couple to the Gq/11 class of G-proteins leading to inositol phosphate (IP) accumulation we have previously observed that JR-6 induced only a weak IP production in CHO expressing M1, and a marginal response in CHO expressing M3 receptors^33^. To examine this signalling, we analyzed the activation of the Gq/11 class of G proteins: Gαq, Gα11, Gα14 and Gα15 upon agonist binding to M1 and M3 using our BRET-based assay. CBC activated all Gq/11-type G proteins with the same magnitude at M1 as well as at M3 receptors except for a slightly higher response of G14 at M1 and a 27% lower magnitude of G15 response at M3 (Fig. 6A, upper part, SI, Table S5 and Fig. S4). Despite the same magnitude of activation of all Gαq/11 types by CBC-activated M1 receptor, their activation rates varied and decreased in order Gq > G11 = G15 > G14 (6A; Table S5). At M3, activation of the Gα15 by CBC was slower than others (Fig. 6A). Compounds JB-8A and JB-13-1 did not activate any Gαq/11 type via M1 or M3 receptors (Fig. 6A, down). Compounds JR-6, PN-152 and JB-12-2 induced activation of Gq/11 types was slower and displayed profoundly distinct signalling patterns from reference agonist CBC (Fig. 6A). Moreover, activation profiles differed between M1 and M3 receptors (Fig. 6B).

Partial agonist JR-6 activated α-subunits G11, Gq and G15 via M1 receptor with magnitude reaching approximately 50% of CBC and weakly activated Gα14 (approx. 10% of CBC) (Fig. 6A, SI Table S5 and Fig. S4). On the other hand, JR-6 induced no response at Gα15, weak response at Gα14 and the magnitude of G11 was smaller than of Gq at the M3 receptor. The highest response of the M3 receptor to JR-6 was measured at Gq (approximately 30% of CBC). The activation rate of Gq, G11 and G14 did not differ at M1 and M3 (Fig. 6A, SI Table S5). The activation rate of Gα15 at M1 was slower than the rate of other Gq/11 types (Fig. 6, Table S5).

Agonist JB-12-2 activated all Gαq/11 types to a greater extent than JR-6. The activation pattern (magnitude ratio among Gα and kinetics) was similar to JR-6. But unlike JR-6, JB-12-2 activated the Gα15 via the M3 receptor (Fig. 6).

PN-152 activated the Gq/11 types to a smaller extent than the JR-6. At the M1 receptor, PN-152 activated Gαq, Gα11 and G15 equally (approx. 30% of CBC), with a slower rate at G15, but did not activate Gα14. At M3, PN-152 induced response only at Gαq which was very weak (10% of CBC) (Fig. 6, SI Table S5).

Moreover, at the M3 receptor, while reference agonist CBC induced activation of non-preferential Gi/o G-proteins, THP-based agonists produced no signal at Gi/o via M3 (SI Table S6). Together, these observations indicate that small partial agonists based on THP-moiety are capable of selective activation of individual G-proteins types with a favourable profile discriminating among individual subtypes of muscarinic receptors based on their tissue and subtype selectivity.

### Molecular modelling of compound interactions reveals a potential mechanism for bias among Gαi/o types

To obtain insights into the structural basis of observed selectivity, we docked parental compound JR-6 and its derivates in the orthosteric binding site of the M2 receptor in an active conformation (4MQS) followed by molecular dynamics simulation experiments. Except for JB-8A and JB-12 compounds, ligands possess chiral nitrogen. Molecular modelling was performed for both S and R enantiomers. According to docking, stereoisomers yielded similar binding energies, S isomers being up to 0.48 kcal/mol higher. Over S isomers, all R isomers employ W155 in TM4, except the R isomer of PN-152 which employs F195 in TM5. W155 and F195 are, however, adjacent residues. Binding energies and principal receptor-ligand interactions are listed in Table 2. Further details are in Supplementary Information Fig. S2.

**Table 2:** Results of docking to the model of the M_2_ receptor (4MQS) in an active conformation.

Compound	Binding energy (kcal/mol)	Principal binding residues										
		TM3	TM3	TM3	TM3	TM4	TM5	TM6	TM6	TM6	TM7	TM7
JR-6 S	6.87	D103	Y104					W400	Y403		Y426	
JR-6 R	6.53	D103	Y104			W155		W400	Y403		Y426	Y430
PN-152 S	6.96	D103	Y104					W400	Y403			Y430
PN-152 R	6.51	D103	Y104				F195	W400	Y403		Y426	Y430
JB-12-1	5.52	D103	Y104					W400	Y403		Y426	Y430
JB-12-2	5.92	D103	Y104			W155		W400	Y403		Y426	Y430
JB-13-1 S	7.54	D103^a^	Y104	S107				W400		N404	Y426	Y430
JB-13-1 R	7.45		Y104	S107	V111	W155			Y403	N404	Y426	
JB-13-2 S	7.92		Y104			W155		W400	Y403	N404	Y426	
JB-13-2 R	7.44		Y104		V111	W155		W400	Y403	N404	Y426	Y430
JB-8A	7.11	D103	Y104			W155		W400	Y403			Y430

Only principal residues interacting with a ligand for more than 30% of the simulation time are shown.

^a^Water bridges.

**Figure 7:**
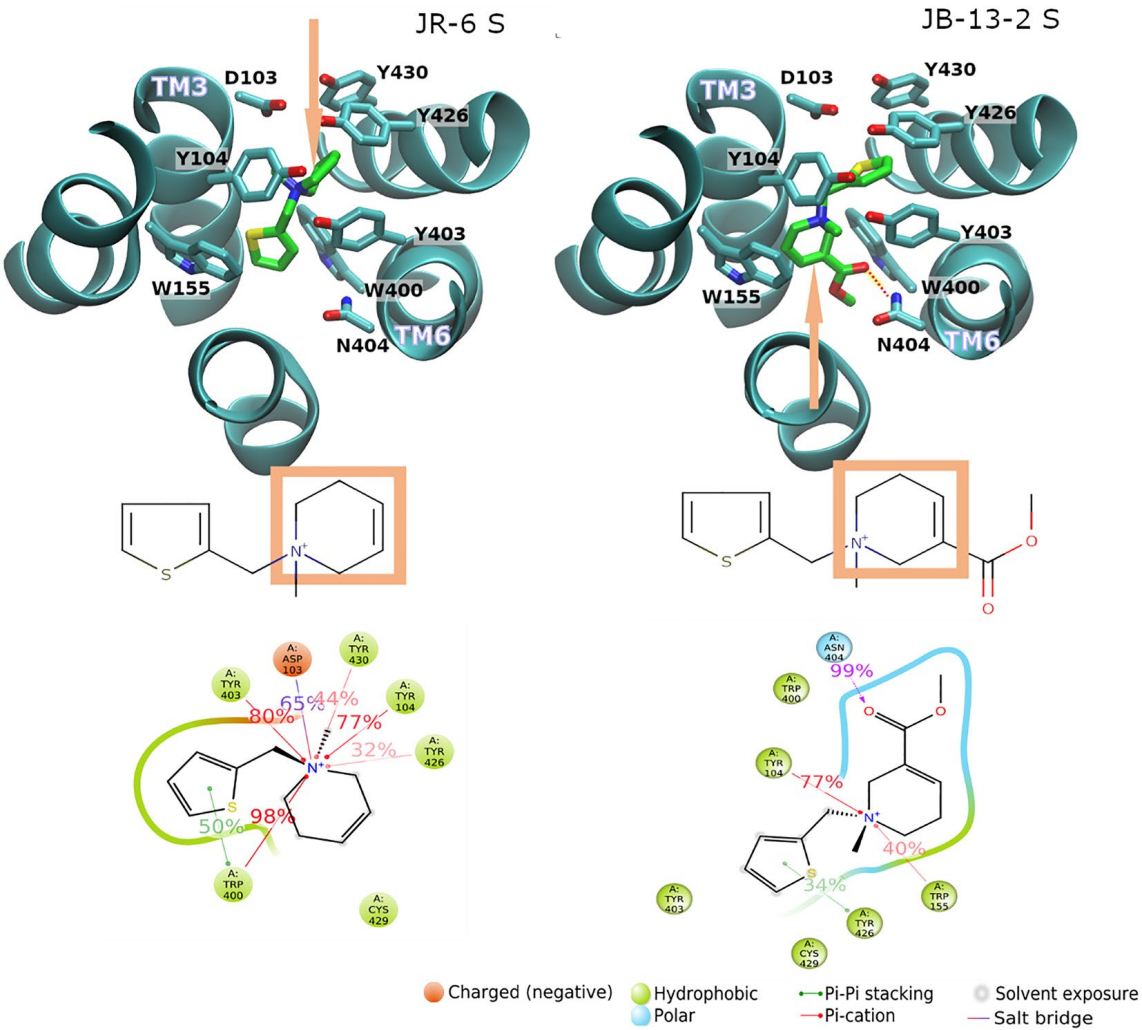
Binding of JR-6 and JB-13-2 in the orthosteric binding site of M_2_ receptor. The model of the M_2_ receptor in an active conformation (4MQS) was used. Top: reversed orientation of R- enantiomers of compounds JR-6 (left) and JB-13-2 (right) is shown. Orange arrows indicate the position of the THP. The hydrogen bond between N404 at TM6 and the carbonyl oxygen of JB-13-2 is shown as red dots. Bottom: schematic of detailed JR-6 (left) and JB-13-2 (right) interactions with the protein residues. Interactions that occur more than 30% of the simulation time of molecular dynamics trajectory are shown.

Predicted binding energies of parental compounds JR-6 and PN-152 were similar, sharing common interactions with D103, Y104 in TM3, W400 in TM6 and one or more of tyrosines Y403, Y426 and Y430 (Table 2). However, the experimental binding affinity of PN-152 was ten-times higher than JR-6 (Table 1). Both compounds lack hydrogen bonding to N404 in TM6, common for classical agonists (e.g., iperoxo in 4MQS) and antagonists (e.g., QNB (1-azabicyclo[2.2.2]octan-3-yl hydroxy(diphenyl)acetate) in 3UON). Instead, they bind deeper in the binding pocket and their thiophene ring forms π-π stacking interaction and nitrogen forms a cation-π interaction with W400 (Fig. 7 left). Common interactions of both compounds with the M2 receptor are in accordance with their similar coupling profile with Gαi/o types at M2 (Fig. 3) and exclusive activation of Gαo only. Replacing the THP ring with quinuclidinyl moiety (JB-8A) resulted in a small increase in the estimated binding energy (Table 2) which is in accordance with an increase in the experimental binding affinity (Table 1). Interaction with principal residues in the orthosteric binding site of the M2 receptor (Fig. S2B), as well as the exclusive coupling to Gαo type, remained the same (Fig. 3). Replacing the THP ring with the morpholine ring (JB-12-1 and JB-12-2) resulted in a small decrease in the predicted binding energy (Table 2), corresponding with a decrease in experimental binding affinity (Table 1). The principal binding residues remained the same (Table 1, Fig. S2B). Although the GTPγ[^35^S] binding assay revealed also weak activation of Gαi types by JB-12-2, this compound was biased to GαoA type (Fig. 3).

The addition of methyl ester group (JB-13-1 and JB-13-2) increased predicted binding energy (Table 1). However, the experimental binding affinity to the M2 receptor decreased (Table 1). This modification changed the orientation of the ligands in the orthosteric binding site (Fig. 7, right). Similarly to classic agonists, the carbonyl oxygen of the compound interacts with N404 in the TM6 via a hydrogen bond. This strong hydrogen bonding replaced relatively weak hydrophobic interactions between the thiophene ring and W400, neighbouring N404, resulting in higher predicted binding energies. In contrast with JR-6 which activated Gαo only, JB-13-1 activated all Gαi/o types via the M2 receptor, being biased to the Gαi3 type. It suggests that interaction with N404 facilitates the activation of Gαi types at M2. Overall, these observations support the notion that even small differences in the structures of agonists can lead to distinct agonist-specific conformations of receptor^34^ and the activation of different sets of signalling pathways.

## Discussion

The main finding of our study is that partial muscarinic agonist JR-6^33^ and its analogues exert specific signalling profiles of coupling with individual types of G-protein α-subunits. These signalling profiles profoundly differ from the reference agonist CBC. Moreover, coupling with individual Gα types induced by these novel agonists varies among subtypes of muscarinic receptors. These findings enrich our understanding of mechanisms of pathway-specific activation revealing subtype and pathway-specific structure-activity relationships of these compounds that can lead to the development of subtype-selective agonists.

The conserved nature of the orthosteric binding site among individual subtypes of muscarinic receptors complicates the development of subtype-selective agonists. The binding of an agonist to one or a subset of functional hot spots within the binding site results in the activation of a subset of signalling pathways and thus in ligand-mediated signalling bias^32^. Agonists small in size have a better chance of interacting with a limited set of functional hotspots. In general, agonists interacting with a smaller number of hot spots possess lower potency and efficacy. On the other hand, they have a potential for pathway-specific activation. However, in areas with high receptor reserve, partial agonists could exert sufficient efficacy and significant pharmacological relevance^42^. In native systems, signalling is often improved by oligomerisation and by the formation of signalosomes where multiple signalling proteins are tightly integrated^43^ which increases the efficacy and rates of agonist-induced signalling^44,45^.

In our recent study, we detected non-uniform signalling of compound JR-6^33^. Using CBC as a reference agonist, JR-6 inhibited cAMP level (Gi/o mediated pathway) at M2 and M4 more than an enhanced accumulation of inositol phosphates (Gq/11 mediated pathway) at M1 and M3. It was demonstrated in overexpressed systems of CHO cells as well in native systems predominantly expressing given muscarinic subtypes. Based on the results from these radiochemical accumulation assays and the application of bias analysis according to Kenakin et al^39^, JR-6 can be considered cAMP (Gi/o) pathway biased and M2 selective partial agonist^33^.

In the present study, we analyzed the signalling profile of the M2 receptor with individual Gαi/o types as well as signalling profiles of M1 and M3 receptors with individual Gαq/11 after stimulation by JR-6 and its structural analogues to get a deeper insight into this non-uniform signalling.

For profiling of Gαi/o types, we used GTPγ[^35^S] binding assays with insect cells recombinantly expressing the M2 receptor in combination with the Gαi/o of interest. Endogenous Gαi-like proteins found in insect cells do not couple productively to the mammalian type of GPCRs^37^. Thus, this highly sensitive system enables analysis of the direct activation of individual Gαi/o following M2 activation by partial agonists exerting low efficacy. Assayed system and method substantially affect observed response to a given agonist. To precisely quantify agonism and discriminate ligand bias from possible system bias data were evaluated according to Kenakin^38^, Kenakin et al.^39^, Grifin et al.^40^ In this procedure the reference agonist serves as an internal standard of the system to discriminate ligand bias from possible system bias. Ligand exerting profound activity at one pathway and no activity at another is deemed to possess absolute signalling selectivity.

Importantly, in this system, reference agonist CBC displayed comparable efficacy at all Gαi/o facilitating our analysis. Surprisingly, JR-6 and its analogues display signalling profiles profoundly different from the reference agonist CBC. The signalling profiles of these analogues also vary substantially. While JR-6, PN-152 and JB-8A activated GαoA and GαoB only, JB-12-2 and JB-13-1 activated all Gαi/o. JB-12-2 was slightly biased toward GαoA. JB-13-1 was slightly biased to Gαi3 (Fig. 4). To determine whether profoundly different signalling profiles in Gαi/o activation is a property of weak THP-based muscarinic agonists or whether it is common among structurally diverse agonists, we analyzed the signalling profiles of five structurally diverse muscarinic agonists exerting distinct potency and efficacy and calculated signalling bias relative to carbachol. For this analysis, we have chosen superagonists iperoxo, classical muscarinic agonists oxotremorine and arecoline and atypical agonists NDMC and Mc-Neil A343. All these agonists activated all Gαi/o (Fig. 2) with various operational efficacies (SI Table S1). As indicated by bias plots (Fig. 3), in comparison with THP-based compounds, the coupling profile of this set of control agonists did not exert as pronounced signalling bias among Gαi/o (Fig. 7), indicating the uniqueness of THP-based agonists regarding the activation of Gαi/o types.

Variations in detected bias were found among classic as well as THP-based agonists (e.g. JR-6 and PN-152 are biased to GαoB, JB-13-1 to Gαi3. Such variations indicate that putative system bias towards GαoA (inferred from functional response to carbachol) was successfully discerned by applied procedures.

At the M2 receptor, JR-6, its chlorinated analogue PN-152 and quinuclidinyl containing JB-8A activated only Gαo (Fig. 2). According to molecular modelling, these compounds do not form a hydrogen bond with N404 (Table 2, SI Fig. S1) present in all X-ray and cryo-EM structures of muscarinic receptors. These agonists bind deeper in the binding pocket and instead of hydrogen bonding to N404, they form π-π interaction between the thiophene ring and W400. Compounds JB-12-2 having morpholine group and methyl ester-substituted JB-13-1 activated all tested Gαi/o types. Molecular modelling shows that the binding of JB-12 is very similar to JR-6. JB-12-2 exerted bias towards GαoA but also activated all tested Gαi/o, indicating the model is low in detail. In contrast, binding JB-13 is reversed in the binding site and forms a hydrogen bond between the carbonyl oxygen and N404. So activation of all tested Gαi/o and bias towards Gαi3 is not surprising. The interaction with N404 may be a potential hot spot for bias towards Gαi types at M2. The hydrogen bond is supposed to have a major contribution to the binding energy (Table 2). Therefore, the model predicted improved binding affinity over JR-6. However, the experimentally determined affinity of JB-13 was lower than the affinity of JR-6 (Table 1). Even small differences in the structures of agonists can lead to distinct agonist-specific conformations of the receptor^34^. Therefore, discrepancies between model predictions and experimental results may be due to ligand docking to the receptor in the conformation that is specific to superagonist iperoxo (4MQS).

Our results demonstrate partial agonism and a unique signalling profile of JR-6 and its analogues at the M2 receptor. In contrast, Jiang et al. using CHO cells expressing the M2 receptor detected an agonistic activity of JR-6 only after a profoundly increasing expression level of the M2 receptor^46^. Cellular background such as receptor expression level and expression of individual G-protein types as well as other downstream cellular elements can influence the apparent signalling efficacy of agonists. Analysis of low-efficacy agonists in cell lines requires using a highly sensitive system. In the present study, we use a very high-expression baculovirus/Sf9 insect cell system of a given pair receptor—Gα and direct analysis of the coupling. In our recent study^33^, we successfully measured the agonistic activity of JR-6 (7A) in CHO cells with high expression levels (10× more than in the study of Jiang et al.)^46^. Importantly, the agonistic activity of JR-6 has also been detected in ex vivo tissues that express muscarinic receptors at relatively low levels but where the high organization of subcellular players boosts agonist efficacy and kinetics^43^.

Muscarinic agonists displaying non-uniform activation of individual Gi/o types offer interesting pharmacological potential. Inhibitory G-protein α-subunits Gi1, Gi2, and Gi3 are widely expressed in the whole body, while the Gαo is predominantly expressed in the brain, especially in the hippocampus, striatum and cerebellum^15–17^. The distribution of Go in the periphery is minimal^47^. Go is one of the most abundant G-proteins in the brain and critical for nervous system function. Studies of mice lacking the Goα-subunit have reported several neurological deficits, including tremors, seizures, repetitive turning behaviour, abnormal exploratory behaviour or poor motor coordination and exhibit hyperalgesia when subjected to a hot plate test^18^. Selective modulation of the M2 receptor in CNS by agonists biased to centrally expressed Gαo type of inhibitory Gi/o-proteins can play a role in analgesia without adverse effects, mediated mainly by activation of peripheral muscarinic receptors such as bradycardia, diarrhoea, sialorrhea etc.

BRET-based real-time monitoring of G-protein activation assay was not sufficiently sensitive to analyse activation of Gαi 1, 2, 3, after receptor stimulation by low efficacy ligands. However, it demonstrated that activation kinetics of Go G-proteins by THP-based compounds JR-6, JB-12-2 and JB-13-1 is very slow in comparison to CBC (Fig. 4, SI Table S4). BRET-based real-time monitoring of G-protein activation was suitable for the analysis of the activation of all Gαq/11 types after the activation of the M1 and M3 receptor even those induced by low-efficacy THP-based agonists. We detected a distinct Gαq/11 activation profile of JR-6 and its analogues from reference agonist CBC. Moreover, signalling patterns of THP-based agonists varied among M1 and M3 receptors. Importantly, the reference agonist CBC activated all Gq/11 types similarly, which made the analysis easier. Interestingly, compound JB-13-1 inducing activation of Gαi/o after stimulation of the M2 receptor (detected by GTPγ[^35^S] binding as well as BRET monitoring) did not activate any Gq/11 type at M1 nor M3 receptor. Reverse orientation of this JR-6 analogue and different binding mode probably leads to activation of Gi/o-specific hotspots of the M2 only and thus despite its low efficacy represents a possible structural hit for the M2 selective agonists.

Treatment of attenuated M1 transmission and subsequent cognitive deficit by non-selective muscarinic agonists induce severe gastrointestinal side effects mediated mainly by activation of M3 receptors^9,10^. Discrimination in activation of individual Gq/11 types between M1 and M3 subtypes may lead to desired M1 vs. M3 selectivity. Our data have demonstrated a remarkable difference in the activation of the Gα15 type between the M1 and the M3 receptors (Fig. 5; SI Table S5). In general, all THP-based agonists activated all Gq/11 types at M3 to a substantially smaller extent than at M1. It is in agreement with our previous results from the measurement of JR-6-induced inositol phosphates accumulation via M1 in comparison to M3 receptors in CHO cells^33^.

Interestingly, while CBC activated Gα14 to a similar extent as other Gq/11 types, THP-based agonists activated this Gα negligibly (Fig. 5; SI Table S5). The agonistic activity of JR-6 at Gα15 at the M1 receptor contrasts data obtained with a fusion protein consisting of the M1 receptor and Gα15 where no accumulation of inositol phosphates was detected after stimulation by JR-6^48^. This can be explained by reliance on different assays, cell lines and the very slow kinetic of JR-6 demonstrated in BRET-based real-time monitoring of Gα15 activation. Moreover, receptor-Gα fusion can itself negatively influence receptor activation induced by low-efficacy agonists, despite an increase in operational efficacy observed at stronger agonists. However, BRET-based monitoring developed by Masuho et al.^41^ uses intact receptor and Gα subunit as well.

Tissue-specific distribution of several Gq/11 class G proteins is well described^20,22^. Therefore, distinct activation of individual Gαq/11 by pathway-specific compounds leading to tissue-specific activation of muscarinic receptors is expected. Biased agonists have the potential to promote therapeutically desired signalling while avoiding signalling pathways associated with detrimental side effects. However, the structural basis of biased agonism leading to selective GPCR signalling has only begun to be understood recently and the design of such ligands has yet to be developed. It was demonstrated that bitopic ligands which bind simultaneously to both highly conserved orthosteric and allosteric binding sites, located on less conserved extracellular loops, can activate distinct subsets of downstream effectors and display biased properties. Thomas et al. have shown, that M1-functionally selective bitopic agonists, AC-42 and 77-LH-, in contrast to balanced orthosteric agonists oxotremorine, arecoline and pilocarpine, activated only Gq/11 and Gs pathways but did not activate Gi/o in CHO cells expressing human M1 receptors^49^. Bock et al. have demonstrated that the bitopic agonist iper-6-naph, unlike orthosteric agonist iperoxo, displays a significant preference for Gi/o activation over Gs activation in CHO cells stably expressing recombinant M2 receptor^50^. Masuho et al.^2^ have revealed a completely different coupling profile of a highly selective M1 bitopic agonist TBPB and orthosteric balanced agonists acetylcholine and oxotremorine to individual G-protein types at M1 receptor expressed in HEK cells. Unlike acetylcholine and oxotremorine, TBPB completely failed to support the activation of inhibitory G-proteins Gi1, Gi2, Gi3 and Go. However, TBPB still efficiently activated Gq, G11, G14 and G15 proteins^2^. Different activation profiles of agonist/partial agonist methacholine and pilocarpine in activation of individual Gαi types for M2 and M4 receptors with marked differences in activation of Gαi types and Gαq/11 for M1 and M3 receptors were described by Akam et al.^51^.

Although the compounds that we describe in this study are weak partial agonists whose characterization required the use of overexpression systems, we believe that such studies reveal unique signalling mechanisms of these compounds which lead to the development of highly efficient optimized compounds with pathway-specific and selective action.

## Materials and methods

### Generation of recombinant baculovirus

Baculovirus/*Spodoptera frugiperda* (Sf9)-insect cells expression system represents a well-defined system enabling recombinant expression of a combination of given GPCR with individual G-protein α-subunits in insect cells at a high level and without contamination by interfering GPCRs and with a limited set of endogenous G-proteins^52^.

Baculoviral stocks for expression of M2 receptor and individual Gα-subunits of Gi/o G-proteins (GαoA, GαoB, Gαi1, Gαi2, Gαi3) in SF9 cells were generated according to Bac-to-Bac^®^ baculovirus expression system—user guide (https://assets.thermofisher.com/TFS-Assets/LSG/manuals/MAN0000414_BactoBacExpressionSystem_UG.pdf). Briefly, plasmids pcDNA3.1 coding human receptors M2 and human Gα subunits of individual Gi/o G-proteins obtained from Missouri S&T cDNA resource center (Rolla, MO, USA) were subcloned into the pFastBac1 donor plasmid (Thermo Fisher Scientific; LOT: 2065047) using restriction endonucleases. pFastBac constructs were transformed into MAX Efficiency^®^ DH10Bac^™^ competent E. coli (Thermo Fisher Scientific; LOT: 2443297) to generate a recombinant bacmid. Bacmid DNA was transfected into adherent insect cell line Sf9 in the 12.5 cm^2^ flask in amount 1.5 × 10^6^ cells per flask in 3 ml of Grace’s unsupplemented medium (Gibco; LOT: 2083249) using 10 µl of Cellfectin reagent (Thermo Fisher Scientific; LOT: 2094064) and 1.3 µg bacmid DNA. After 96 h at 27 °C, baculoviral viral particles released into the medium were harvested. After centrifugation at 500×*g* for 10 min to remove detached cells and cell debris, baculoviral stock P0 was ready for amplification. Suspension of Sf9 cells at density 2 × 10^6^ cells per ml was infected by baculoviral stock P0 in ratio 1:1000 and after 72 h of growth (in a shaking incubator at 135 rpm and 27 °C) the amplified baculoviral stock P1 was harvested. The cell suspension was centrifugated at 500×*g* for 10 min to remove cells and cell debris and the supernatant containing a high level of baculoviral particles P1 was collected. FBS to the concentration of 0.5% was added and the stock was stored at 4 °C, protected from light for up to 6 months. For the quantification of baculoviral particles, a plaque assay was used. The titer of baculoviral stock was 3–9 × 10^7^ pfu/ml (plaque forming unit per ml).

### Cell culturing and transfection

*CHO cells* stably transfected with the genes of individual subtypes of human muscarinic receptors (M1–M5, wild types) were purchased from Missouri S&T cDNA resource center (Rolla, MO, USA) (https://www.cdna.org/home.php?cat=177). Cells were cultured as described previously by Randakova et al.^33^. Cells were grown to confluence in 75 cm^2^ flasks in Dulbecco’s modified Eagle’s medium DMEM (Thermo Fisher Scientific; LOT: 2556745) supplemented with 10% fetal bovine serum and geneticin in concentration 50 µg/ml at 37 °C in a humidified incubator containing 5% CO_2_. The medium was supplemented with 5 mM butyrate for the last 24 h of culture to increase receptor expression. Cells were washed with (phosphate-buffered saline (PBS), mechanically harvested by scraper and centrifugated 1000 × *g* for 5 min. Cell pellets were kept at − 80 °C.

*HE/K293T17* cells were obtained from The Global Bioresource Center ATCC (Manassas, VA, USA). Cells were grown in 100 mm petri dishes in Dulbecco’s modified Eagle’s medium (DMEM) supplemented with 10% fetal bovine serum (FBS), MEM non-essential amino acids, 1 mM sodium pyruvate, and antibiotics (100 units/ml penicillin and 100 μg/ml streptomycin) at 37 °C in a humidified incubator containing 5% CO_2_^41^*.* Transfection was performed according to Masuho et al.^41^. Culture dishes (3.5 cm) were coated by incubation for 10 min at 37 °C with 1 ml of Matrigel solution [approximately 10 μg/ml of growth factor-reduced Matrigel (BD biosciences; LOT: 2278001) in culture medium]. For transfections, cells were seeded in the 3.5 cm dishes containing the Matrigel solution at a density of 1.7 × 10^6^ cells/dish. Four hours later, the cells were transfected with the appropriate expression constructs (total of 5 μg DNA per dish) with the reagents PLUS (5 μl/dish) and Lipofectamine LTX (Thermo Fisher Scientific; LOT: 2640614) (6 μl/dish). The cells were transfected with the Venus 156-239-Gβ1 (0.21 μg), Venus 1–155-Gγ2 (0.21 μg), and masGRK3ct-Nluc (0.21 μg) constructs in addition to the different amounts of constructs for the M2 receptor and Gα of interest. Gα14 subunit was co-transfected with Ric-8A chaperone (0.21 μg). Cells were cotransfected with a pcDNA3.1 construct encoding the catalytic subunit of pertussis toxin (PTX-S1) (0.21 μg) and constructs encoding Gα15, Gα14, Gα11, Gαq to ensure that the small BRET signals were not contaminated by the possible recruitment of endogenous Gαi/o proteins. The empty vector pcDNA3.1 was used to normalize the amount of DNA in each transfection.

*Sf9 cells* (Gibco™ Cat. No. 12659017) were purchased from Thermo Fisher Scientific (Waltham, MA, USA) and maintained according to provider guidelines (https://assets.thermofisher.com/TFS-Assets/LSG/manuals/Sf9_SFM_II_SFM_III_man.pdf). Cells were grown in suspension culture in 250 ml Erlenmeyer flasks in Sf-900^™^ III serum-free medium in an aerated shaking incubator at 27 °C and 135 rpm. Cells were maintained at the density of 1–4 × 10^6^ cells per ml. Sf9 cells at the density 2 × 10^6^ were co-infected with baculoviral stock for the M2 receptor and a particular Gi/o Gα in the ratio (1:10). The endogenous Gβγ subunits were sufficient for the ^35^S-GTPγS assay. Infected cells were harvested by centrifugation 1000×*g* for 10 min. Cell pellets were kept at − 80 °C.

All cell lines used in this study present the characteristic morphology. All cell lines were authenticated by the provider and were cultured according to the protocol from the providers, and no further authentication procedure was performed. All the cell lines used in this study are free of mycoplasma contamination.

### Membrane preparation

Membranes were prepared as described previously by Randakova et al.^33^. Cells harvested from twenty 100 mm petri dishes (CHO) or 30 ml of cell suspension (Sf9) were suspended in 20 ml of ice-cold incubation medium (100 mM NaCl, 20 mM Na-HEPES, 10 mM MgCl_2_, pH  7.4) supplemented with 10 mM EDTA and homogenized on ice by two 30‐s strokes using a Polytron homogenizer (Ultra-Turrax; Janke & Kunkel GmbH & Co. KG, IKA-Labortechnik, Staufen, Germany) with a 30 s pause between strokes. Cell homogenates were centrifuged for 5 min at 1000×*g* to remove whole cells and cell nuclei. The resulting supernatants were centrifuged for 30 min at 30,000×*g*. Pellets were suspended in a fresh incubation medium, incubated on ice for 30 min, and centrifuged again. The resulting membrane pellets were kept at − 80 °C until assayed within 10 weeks.

### Determination of affinity of tetrahydropiridine-based agonists to muscarinic receptors in membranes

The affinity of novel agonists to individual subtypes of muscarinic receptors (M1–M5) was determined in competition binding experiments with 0.5 nM radiolabelled non-selective muscarinic antagonist [^3^H]N-methylscopolamine (NMS) (PerkinElmer; LOT: 210212) as described by El-Fakahany & Jakubik^53^. Briefly, cell membranes from CHO cells stably expressing individual subtypes of muscarinic receptors, approximately 10 μg of membrane protein per sample, were incubated in 96-well plates at 30 °C in the incubation medium described above in incubation volume 400 μl in presence of [^3^H]NMS and increasing concentration of tested agonist for 1 (M2), 3 (M1, M3, and M4), or 5 h (M5). Non-specific binding was determined in the presence of 10 μM unlabelled atropine. Incubations were terminated by filtration through filtration plates multiscreen 96 well Harvest (Merck Millipore) using a Brandel cell harvester (Brandel, Gaithersburg, MD, USA). Filtration plates were dried in a microwave oven, and then 40 μl of liquid scintillator Rotiscint eco plus (Roth) was added. The filtration plates were counted in the Microbeta scintillation counter (PerkinElmer). Concentration causing 50% inhibition of radioligand binding IC50 was determined according to Eq. (1) after subtraction of non-specific binding. The inhibition constant KI was calculated according to Eq. (2).

### GTPγ[^35^S]binding in membranes

Agonist stimulated GTPγ[^35^S]binding to membranes from Sf9 cells expressing the M2 receptors in a combination with α-subunit of given Gi/o type (GαoA, GαoB, Gαi1, Gαi2, Gαi3) was measured in 96-well plates in a final volume of 200 µl of incubation medium described above containing 500 pM of GTPγ[^35^S] (PerkinElmer; LOT: 1122) and 20 µM GDP for 20 min at 30 °C after 15 min preincubation with GDP and agonist. The concentrations of agonists up to 100 μM for JB-8A, JR-6 and PN-152 and up to 1 mM for JB-12-2 and JB-13-1, corresponding to 10–100 fold of their Ki value, were used. Nonspecific binding was determined in the presence of 1 µM nonlabelled GTPγS. Incubations were terminated and processed as described above. Parameters of functional response were calculated according to Eq. (3) after subtraction of non-specific binding.

### Monitoring G-protein activation in intact cells

To examine the activation of G-protein signalling in live cells, agonist-dependent cellular measurements of BRET between Venus-Gβ1γ2 and masGRK3ct-Nluc were performed as described by Masuho et al.^41^. The saturated concentrations of given agonists (100 μM for PN-152, JB-8A and JR-6 and 1 mM for JB-12-2 and JB-13-1) were used. 18 h after transfection, HEK293T/17 cells were washed once with BRET buffer (phosphate-buffered saline (PBS) containing 0.5 mM MgCl_2_ and 0.1% glucose) and detached by gentle pipetting over the monolayer. Cells were harvested by centrifugation at 500×*g* for 5 min and were resuspended in BRET buffer. About 50,000 to 100,000 cells per well were distributed in 96-well flat-bottomed white microplates (Greiner Bio-One) in a volume of 25 µl. 25 µl of 2× luciferase substrate: Nano-Glo^™^ luciferase assay substrate (Promega; LOT: 0000527335) dissolved in 250 volumes of BRET buffer was added. BRET measurements were made with a microplate reader (POLARstar Omega, BMG Labtech) equipped with two emission photomultiplier tubes, which enabled the detection of two emissions simultaneously with the highest possible resolution of 20 ms per data point. All measurements were performed at room temperature. The BRET signal was determined by calculating the ratio of the light emitted by Venus-Gβ1γ2 (535 nm with a 30 nm band path width) to the light emitted by masGRK3ct-Nluc (475 nm with a 30 nm band path width). The average baseline value (basal BRET ratio) recorded before stimulation of cells with an agonist was subtracted from the experimental BRET signal values to obtain the ΔBRET ratio.

### Molecular modelling

#### Preparation of receptor structure

The structure of the M_2_ muscarinic acetylcholine receptor in an active state 4MQS^54^ was downloaded from the RCSB Protein Data Bank (https://www.rcsb.org/). Non-protein and nanobody molecules were deleted, and the resulting receptor protein was processed in Maestro using Protein Preparation Wizard according to Sastry et al. guidelines^55^.

#### Ligand docking

Docking of muscarinic agonist to 4MQS structure was done using YASARA^56^ implementation of AutoDock^57^. The agonists were constructed in ChemAxon MarvinSketch, parametrized and energy minimized in YASARA. The orthosteric binding site was defined as a 5 Å extended cuboid around co-crystallized iperoxo. The agonists were docked to the orthosteric binding site using the AutoDock local search procedure for 888 poses. All poses were energy minimized and rescored using AutoDock VINA’s local search, confined closely to the original ligand pose. The pose with the highest rescore value was selected for further work^58^.

#### Simulation of molecular dynamics

To evaluate agonist binding to the receptor and quantify its interactions with the receptor, conventional molecular dynamics (cMD) was simulated using Desmond ver. 6.8^59^. The simulated system consisted of the receptor-ligand complex in 1-palmitoyl-2-oleoyl-sn-glycero-3-phosphocholine (POPC) membrane set to receptor helices in water and 0.15 NaCl. The system was first relaxed by the standard Desmond protocol for membrane proteins and then 120 ns γNPT (Noose-Hover chain thermostat at 300 K, Martyna–Tobias–Klein barostat at 1.01325 bar, isotropic coupling, Coulombic cutoff at 0.9 nm) molecular dynamics without restrains was simulated. MD was run three times with random initial velocities^58^. The quality of molecular dynamics simulation was assessed by the simulation quality analysis tools of Maestro and analyzed by the simulation event analysis tool. Ligand–receptor interactions were identified using the simulation interaction diagram tool. MD trajectories were analyzed in visual molecular dynamics (VMD) (http://www.ks.uiuc.edu/Research/vmd/)^60^.

#### Data analysis

Data were processed in Microsoft Office, analyzed, and plotted using the program Grace and GraphPad Prism 6. The statistic was calculated using R (www.r-project.org) and GraphPad Prism 6.

#### Competition binding

The binding of the tested ligand was determined in competition experiments with [^3^H]NMS^53^. Eq. (1) for the one-site competition was fitted to the data.

1 $${\text{y = 100}} - \frac{{{100 \times }x}}{{{\text{x + IC}}_{{{50}}} }}$$

where y is specific radioligand binding at concentration *x* of competitor expressed as a per cent of binding in the absence of a competitor. IC_50_ is the concentration causing 50% inhibition of radioligand binding.

Inhibition constants K_I_ were calculated according to Eq. (2).

2 $$K_{I} = \frac{{{\text{IC}}_{{{50}}} }}{{1 + \frac{\left[ D \right]}{{K_{D} }}}}$$

where IC_50_ is calculated according to Eq. (1) from competition binding data, [D] is the concentration of [^3^H]NMS used, and K_D_ is its equilibrium dissociation constant.

#### Functional response

The potency of analyzed agonists (EC_50_) to induce maximal response (Eʹ_MAX_) was obtained by fitting Eq. (3) to the data from GTPγ[^35^S]binding.

3 $${\text{y}} = 1 + \frac{{\left( {{\text{E}}^\prime_{{{\text{MAX}}}} - 1} \right) \times x^{{{\text{nH}}}} }}{{{\text{EC}}_{{{50}}}^{{{\text{nH}}}} + {\text{x}}^{{{\text{nH}}}} }}$$

where y is a functional response expressed as fold over basal after subtraction of non-specific binding at a concentration of tested compound x, E'_MAX_ is the apparent maximal response to the tested compound, EC_50_ is the concentration causing a half-maximal effect and nH is the slope factor (Hill coefficient).

The basal value of GTPγ[^35^S] binding was determined in the absence of the agonist. After subtraction of non-specific binding determined in the presence of 10 μM nonlabelled GTPγS, GTPγ[^35^S]binding was divided by the basal value and expressed as folds over basal. In Fig. 2, all curves start at 1. Then Eq. (3) was fitted to the data using GraphPad. Obtained E_MAX_ and EC_50_ values were used to calculate RAi values using Eq. (7). To determine τ and K_A_ of the functional responses system E_MAX_ was determined as described earlier^61^. Basal values were subtracted and functional responses were expressed as fractions of system E_MAX_. Then, Eq. (4) was fitted to the data with E_MAX_ fixed to 1. Obtained τ and K_A_ values were used to calculate the bias factor according to Eq. (8), see below, for each experiment.

#### The operational model of functional agonism

The operational efficacy coefficient *τ* was determined by fitting Eq. (4) to data from the functional assays^62^.

4 $$y = E_{MAX} \frac{{x^{{n_{H} }} \frac{\tau }{\tau + 1}}}{{x^{{n_{H} }} + \left( {\frac{{K_{A} }}{\tau + 1}} \right)^{{n_{H} }} }}$$

where *y* is a functional response at a concentration of tested compound *x*, *E*_*MAX*_ is the maximal response of the system, *K*_*A*_ is the equilibrium dissociation constant and *n*_*H*_ is the slope factor. Equation (4) was fitted to data by the two-step procedure described earlier^61^ In the first step, system *E*_*MAX*_ was determined using carbachol, oxotremorine, and pilocarpine as internal standards by fitting Eq. (3) to the functional-response data, plotting observed maximal responses to agonists E’_MAX_ as a function of their half-efficient concentrations EC_50_ and fitting Eq. (5) to the data.

5 $${\text{E}}^\prime_{{{\text{MAX}}}} = E_{{{\text{MAX}}}} - \frac{{E_{{{\text{MAX}}}} \times {\text{EC}}_{{{50}}} }}{{K_{A} }}$$

In the second step, Eq. (4) with *E*_*MAX*_ fixed to the value determined in the first step was fitted to individual experimental data sets.

#### Relative intrinsic activity

For comparison of effects of agonists at individual Gi/o Gα on GTPγ[^35^S]binding, relative intrinsic activity (*RAi*) was calculated according to Griffin et al.^40^:

6 $$RA_{i} = \frac{{\tau_{carbachol} { \times }K_{Aa} }}{{\tau_{a} { \times }K_{Acarbachol} }}$$

where *τa* and *K*_*Aa*_ are the operational efficacies and equilibrium dissociation constants of the tested compound, respectively, obtained by fitting Eq. (4) to the functional-response data. As Hill coefficients were equal to one, *RAi* values were calculated according to Eq. (7).

7 $$RA_{i} = \frac{{E^{\prime}_{MAXcarbachol} { \times }EC_{50a} }}{{E^{\prime}_{MAXa} { \times }EC_{50carbachol} }}$$

where *EC*_*50*_*a* and *Eʹ*_*MAX*_*a* are half-effective concentrations and apparent maximal responses to the tested compound, respectively. Relative intrinsic activities were calculated for each pair of ligand and carbachol in each experiment.

#### Quantifiaction of agonist bias

All evaluation is done according to standard procedures for evaluation of biased signalling using CBC as a reference agonist (e.g., Kenakin^38^, Kenakin et al.^39^, Kolb et al.^63^). Operational efficacy (τ) and agonist equilibrium dissociation constant (K_A_) of tested agonist and reference agonist are sufficient to quantify the agonism of a given ligand. The parameter K_A_ is specific to a combination of ligand and receptor. The parameter τ is specific to a combination of ligand and signalling system. A parameter for characterizing agonism for a given system can be defined as a “transduction coefficient” and is equal to the logarithm of τ to K_A_ ratio, log(τ/K_A_). The relative efficiency of two agonists producing activation of a given pathway is quantified as the difference between the value of the transduction coefficient of the tested ligand and the transduction coefficient of the reference ligand, Δlog(τ/K_A_). For a biased ligand, the Δlog(τ/K_A_) of the biased pathway is greater than the Δlog(τ/K_A_) of the reference pathway.

8 $$Bias factor = 10^{{\Delta log\left( {\frac{\tau }{{K_{A} }}} \right)_{G\alpha A} - \Delta log\left( {\frac{\tau }{{K_{A} }}} \right)_{G\alpha B} }}$$

Bias factors were calculated for each pair of ligand and carbachol in each experiment for all combinations of measured signalling pathways.

#### Association kinetics

The ΔBRET ratio from real-time monitoring of G-protein activation was fitted using monophasic association according to Eq. 9.

9 $${\text{Y}} = Beq \times (1 - {\text{exp}}\left( { - Kobs \times x} \right)$$

where y is the ΔBRET ratio of the light emitted by Venus-Gβ1γ2 (535/30) to the light emitted by masGRK3ct-Nluc (475/30). Beq is Y-value at the equilibrium expressing the maximal amplitude of ΔBRET ratio, x is time and Kobs is the observed rate of Gα subunit activation.

### Statistics and reproducibility

GTPγ[^35^S]binding assay was performed in quadruplicates. Dose–response curves were calculated with GraphPad Prism v 6.0.7 using the log(dose) response curve with variable slope. BRET assay was performed in triplicates. Association kinetics was calculated with GraphPad Prism v 6.0.7 using monophasic association. Data are mean ± SD calculated from three independent experiments (SI Tables S1–S6).

### Supplementary Information

Supplementary Information.

## Data Availability

The authors declare that the data supporting the findings of this study are available within the paper and its Supplementary Information files. Should any raw data files be needed in another format they are available from the corresponding authors upon reasonable request.
