# Supplementary material for: Agonist-selective activation of individual G-proteins by muscarinic receptors
Source: Sci Rep. 2024 Apr 26;14:9652. doi: 10.1038/s41598-024-60259-4 (PMC11053168; doi:10.1038/s41598-024-60259-4)
Supplement: Supplementary file 1 — Supplementary Information. [file 41598_2024_60259_MOESM1_ESM.pdf]

Supplementary information

Agonist-selective activation of individual G-proteins by muscarinic receptors

[1] Dominik Nelic, [1] Nikolai Chetverikov, [1] Martina Hochmalová, [2] Christina Diaz, [1] Vladimír Doležal, [2] John Boulos, [1]Jan Jakubík, [3] Kirill Martemyanov✉ and [1,3] Alena Janoušková-Randáková✉

1 Department of Neurochemistry, Institute of Physiology Czech Academy of Sciences, Prague, Czech Republic; 2 Department of Physical Sciences, Barry University, Miami Shores, Florida, USA; 3 Department of Neuroscience, UF Scripps Biomedical Research, University of Florida, Jupiter, FL 33458, USA;

Supplementary figures

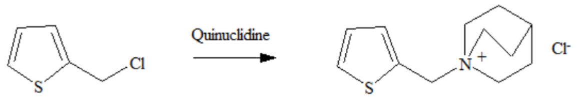

(1)

Figure S1-A: Synthesis of JB-8A (1).

2-(chloromethyl)thiophene: A mixture of 19.0 grams (0.166 mol) of 2-thiophenemethanol, 50.62 grams of triphenylphosphine (0.193 mol) and 120 mL of CCl_4 was refluxed for about 3 hours. About 200 mL of anhydrous pentane was added, and the solution was filtered, concentrated and distilled to afford 8.9 grams (40.5%) of the liquid boiling at 62 °C/14 mmHg. ^{1}H NMR ($CDCl_3$): δ7.4 (1H, d), 7.15 (1H, d), 7.0 (1H, dd), 4.8 (2H, s). **2-(quinuclidinylmethyl)thiophene chloride (1):** 1.0 gram (0.00755 mol) of 2-(chloromethyl)thiophene was added to 0.84 gram (0.00755 mol) of quinuclidine dissolved in 10 mL of acetonitrile. The mixture solidified within minutes, vacuum- filtered and washed with anhydrous ether. The residue was dried to afford 1.55 grams (84%) of **1**, m.p. 257-260 °C. The solid was recrystallized from a mixture of CH_2Cl_2 and anhydrous ether. Dichloromethane was added to dissolve the solid with warming. Ether was then added slowly until the formation of crystals, 1.30 gram, m.p. 257.1- 257.5 °C. ^{1}H NMR (D_2O): δ7.6 (1H, d), 7.5 (1H, d), 7.1 (1H, dd), 5.5 (2H, s), 3.85 (6H, m), 2.2 (1H, m), 2.0 (6H, m).

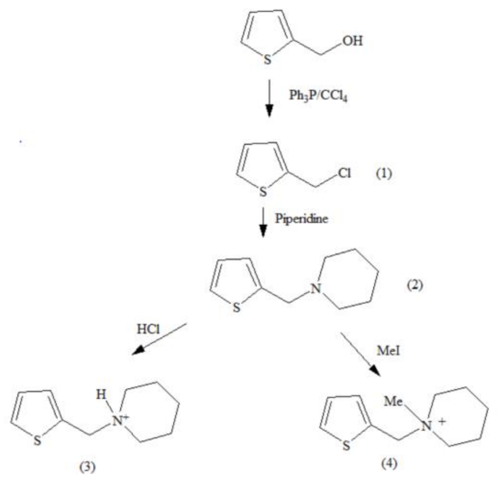

Figure S1-B: Synthesis of JB-11-1 (3) and JB-11-2 (4):

2-(piperidylmethyl)thiophene (2): A mixture of 1.0 g (0.00755 mol) of 2-(chloromethyl)thiophene (1), 0.642 g of piperidine (0.00755 mol), 1.0 g of Na_2CO_3 and 10 mL of acetonitrile was stirred overnight at 60 ^oC. The solution was filtered, concentrated, redissolved in 15 mL of CH_2Cl_2 and extracted with 5 mL of 3M NaOH. The organic layer was washed with 5 mL of H_2O and dried over anhydrous $MgSO_4$, filtered and concentrated to afford 0.71 g (52.6%) of 2. ^1H NMR ($CDCl_3$): δ7.3 (1H, d), 7.00 (1H, dd), 6.95 (1H, d), 3.75 (2H, s), 2.5 (4H, t) 1.65 (4H, m), 1.5 (2H, m). **2-(piperidylmethyl)thiophene hydrochloride (3)**: HCl gas was passed through a solution of 0.58 g of 2 (0.00320 mol) dissolved in 5 mL of acetonitrile for about 2 minutes and then concentrated to afford 0.69 g of 3. The solid was recrystallized from hexanol-ether mixture to yield 0.50 g (71.4%), m. p. 165- 166 ^oC. ^1H NMR (H_2O): δ7.7 (1H, d), 7.2 (1H, d), 7.05 (1H, dd), 4.4 (2H, s), 3.4 (2H, m), 2.9-2.8 (2H, m), 1.8 (2H, m), 1.7 (2H, m), 1.5-1.3 (2H, m). **2-[(methylpiperidyl)methyl]thiophene iodide (4)**: A solution containing 0.71 g (0.00392 mol) of 2, 1 mL iodomethane and 3 mL of acetonitrile was stirred overnight at room temperature. The mixture was concentrated, and the residue was washed with ether and dried to afford 1.2 g of 4. The solid was recrystallized from n-butanol to yield 0.78 g (61.4%) of 4, m. p. 160-161 ^oC. ^1H NMR (H_2O): δ7.6(1H, d), 7.3 (1H, d), 7.1 (1H, dd), 4.6 (2H, s), 3.3 (4H, m), 2.9 (3H, s), 1.8 (4H, m), 1.6 (2H, m)

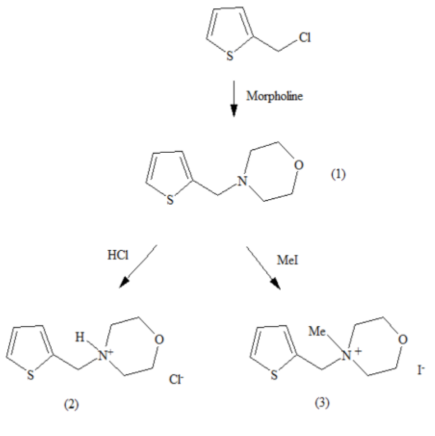

Figure S1-C: Synthesis of JB-12-1 (2) and JB-12-2 (3):

2-(morpholin-4-ylmethyl)thiophene (1): A mixture containing 1.0 g of 2-(chloromethyl)thiophene (0.00755 mol), 0.657 g (0.00755 mol) of morpholine, 1.0 g of Na_2CO_3 and 10 mL of acetonitrile was stirred overnight at 60 ^oC. To the solution was added 25 mL of CH_2Cl_2 and then extracted with 10 mL of 3M of NaOH. The solution was saturated with solid sodium chloride and the top organic layer was dried over $MgSO_4$, filtered and concentrated to yield 1.05 g of 1 (76%).^1H NMR ($CDCl_3$): δ7.3 (1H, d), 7.00 (2H, m), 3.8 (2H, s), 3.8(4H, m), 2.5 (4H, m). **2-(morpholin-4-ylmethyl)thiophene hydrochloride (2)**: HCl gas was bubbled through a solution containing 0.525 g of 1 (0.00287 mol) and 5 mL of acetonitrile for a few minutes. The mixture was concentrated and recrystallized from n-butanol to afford 0.2 g of 2 (33.3%), m.p. 215.4-216 ^oC.^1H NMR (D_2O): δ7.5 (1H, d), 7.25 (1H, d), 7.1 (1H, dd), 4.5 (2H, s), 4.0 (2H, m), 3.7 (2H, m), 3.35 (2H, m), 3.15 (2H, m). **2-[(4-methylmorpholin-4-yl)methyl]thiophene iodide (3)**: About 1 mL of iodomethane was added to a solution of 0.525 g of 1 (0.00287 mol) dissolved in 5 mL of acetonitrile. The mixture was stirred overnight, concentrated and recrystallized from n-butanol to afford 0.77 g (83%) of 3, m.p. 137-138 ^oC. ^1H NMR (D_2O): δ7.6 (1H, d), 7.35 (1H, d), 7.1 (1H, dd), 4.8 (2H, s), 4.1-3.9 (4H, m), 3.6-3.5 (2H, m), 3.4-3.3 (2H, m), 3.1 (3H, s).

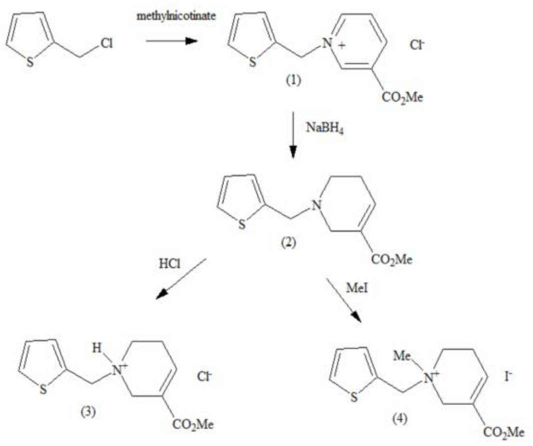

Figure S1-D: Synthesis of JB-13-1(3) and Jb-13-2(4):

methyl 1-(2-thienylmethyl)pyridine-3-carboxylate chloride (1): A mixture containing of 4.0 g of 2-(chloromethyl)thiophene (0.0302 mol), 4.14 g (0.0302 mol) of 4-methyl nicotinate and 20 mL of acetonitrile was stirred for 9 days at room temperature. The mixture was concentrated, and residue washed with anhydrous ether. The crude was dissolved in 1:1 methanol-ether, organic layer was withdrawn and concentrated to afford 8.0 g of 1 (98%), m.p. 112-115 ºC. ^{1}H-NMR (D_2O): δ9.4 (1H, s), 9.0 (1H, d), 8.9 (1H,d), 8.1 (1H,m), 7.5 (1H, d), 7.35 (1H, d), 7.05 (1H, m), 6.0 (2H, s), 3.9 (3H, s). **methyl 1-(2-thienylmethyl)-1,2,5,6-tetrahydropyridine-3-carboxylate (2)**: To a solution containing 8.0 g of 1 (0.0296 m0l) and 45 mL of methanol was added a mixture of 1.5 g (0.04 mol) of $NaBH_4$ in 70 mL of 0.1M NaOH with cooling and stirring. The mixture was stirred for an additional 1 hour at room temperature, acidified (pH 6) and made basic with 3M NaOH (pH 8). The solution was then extracted with CH_2Cl_2, extracts were dried over $MgSO_4$, filtered and concentrated to yield 5.89 g of pasty residue. This residue was chromatographed with anhydrous pentane, orange-colored fraction was collected and concentrated to afford 1.70 g of 2 (24.2%). ^{1}H-NMR ($CDCl_3$): δ7.3 (1H, m), 7.1 (1H, m), 7.0 (2H, m), 3.95 (2H, s), 3.78 (3H, s), 3.3 (2H, m), 2.65 (2H, m), 2.4 (2H, m). **methyl 1-(2-thienylmethyl)-1,2,5,6-tetrahydropyridine-3-carboxylate hydrochloride (3)**: HCl gas was passed through a solution containing 0.300 g (0.00127 mol) of 2 and 4.0 mL of acetonitrile for about 1 minute, concentrated and recrystallized from n-butanol to afford 0.316 g (91%) of 3, m.p. 179-180 ºC. ^{1}H-NMR (D_2O): δ7.5 (1H, d), 7.25 (1H, d), 7.1 (2H, m), 4.6 (2H, s), 3.9 (2H, m), 3.65 (3H, s), 3.5-3.25 (2H, m), 2.1 (2H, m). **methyl 1-methyl-1-(2-thienylmethyl)-1,2,5,6-tetrahydropyridine-3-carboxylate iodide (4)**: About 1.0 mL of iodomethane was added to a solution of 0.45 g (0.00190 mol) of 2 and 5 mL of acetonitrile. The mixture was stirred at RT overnight, concentrated and recrystallized from n-butanol to afford 0.510 g of 4 (71%), m.p. 145.1-145.7 ºC. ^{1}H-NMR (D_2O):): δ7.6 (1H, d), 7.3 (1H, d), 7.2 (1H, m), 7.1 (1H, dd), 4.75 (2H, s), 4.2-4.1(1H, m), 4.0-3.9 (1H, m), 3.7 (3H, s), 3.55-3.35 (2H, m), 2.95 (3H, s), 2.75-2.65 (2H, m).

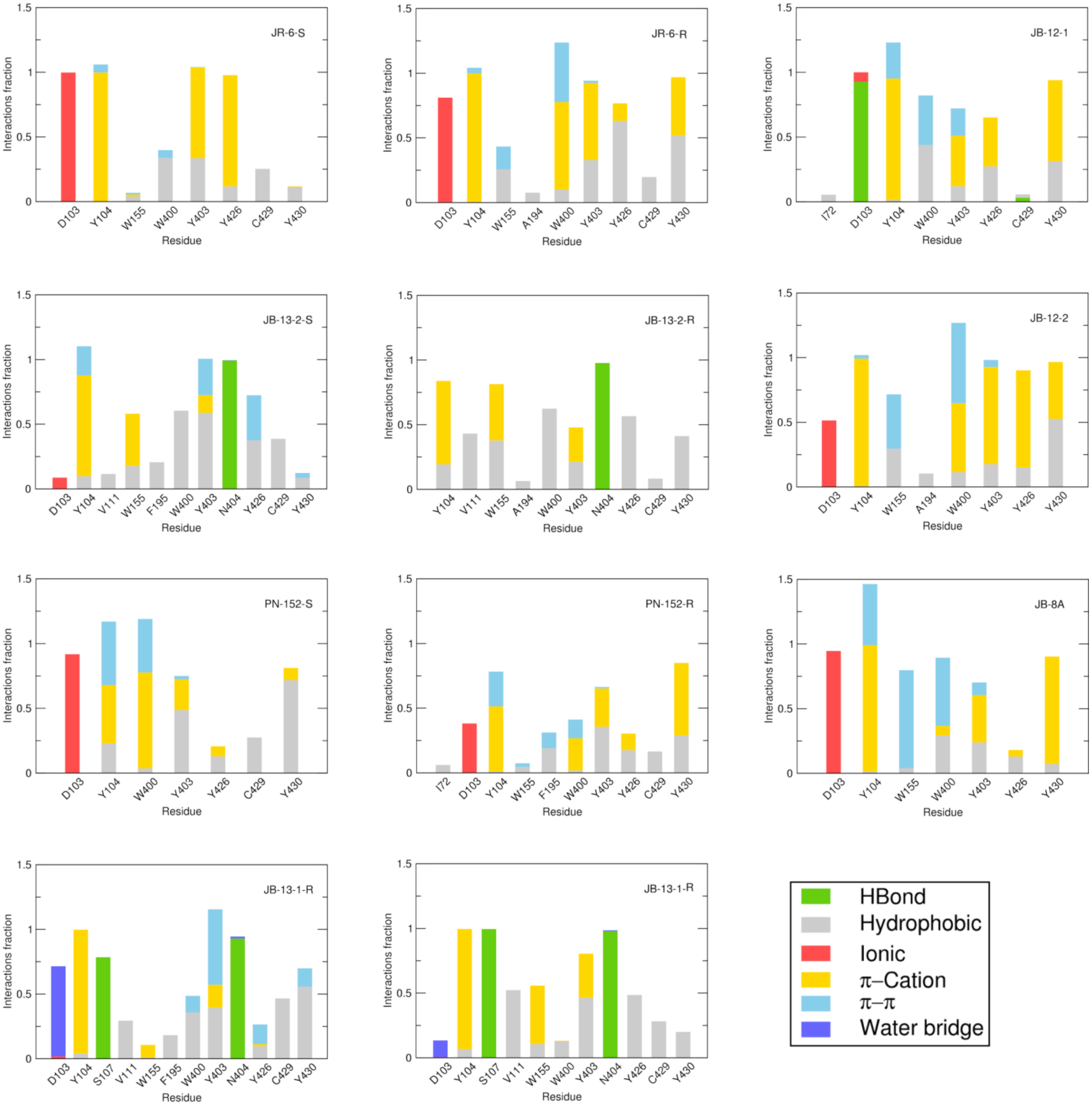

Figure S2A: Interactions between ligands and receptor

Histograms of interactions between agonist (indicated in the legend) and M2 receptor in an active conformation (4MQS) were calculated from MD trajectories using the Ligand Interaction Diagram in Maestro. Only residues interacting with a ligand for more than 5% of time-frames are displayed. The interactions are categorized into six types: Hydrogen bonds (green), hydrophobic (grey), ionic (red), π-cation (yellow), π-π (cyan) and water bridges (blue). The stacked bar charts are normalized over the course of the trajectory. Values are means of 3 runs. SD < 5% points. Values over 1.0 are possible as some protein residues may make multiple contacts with ligands within one time frame.

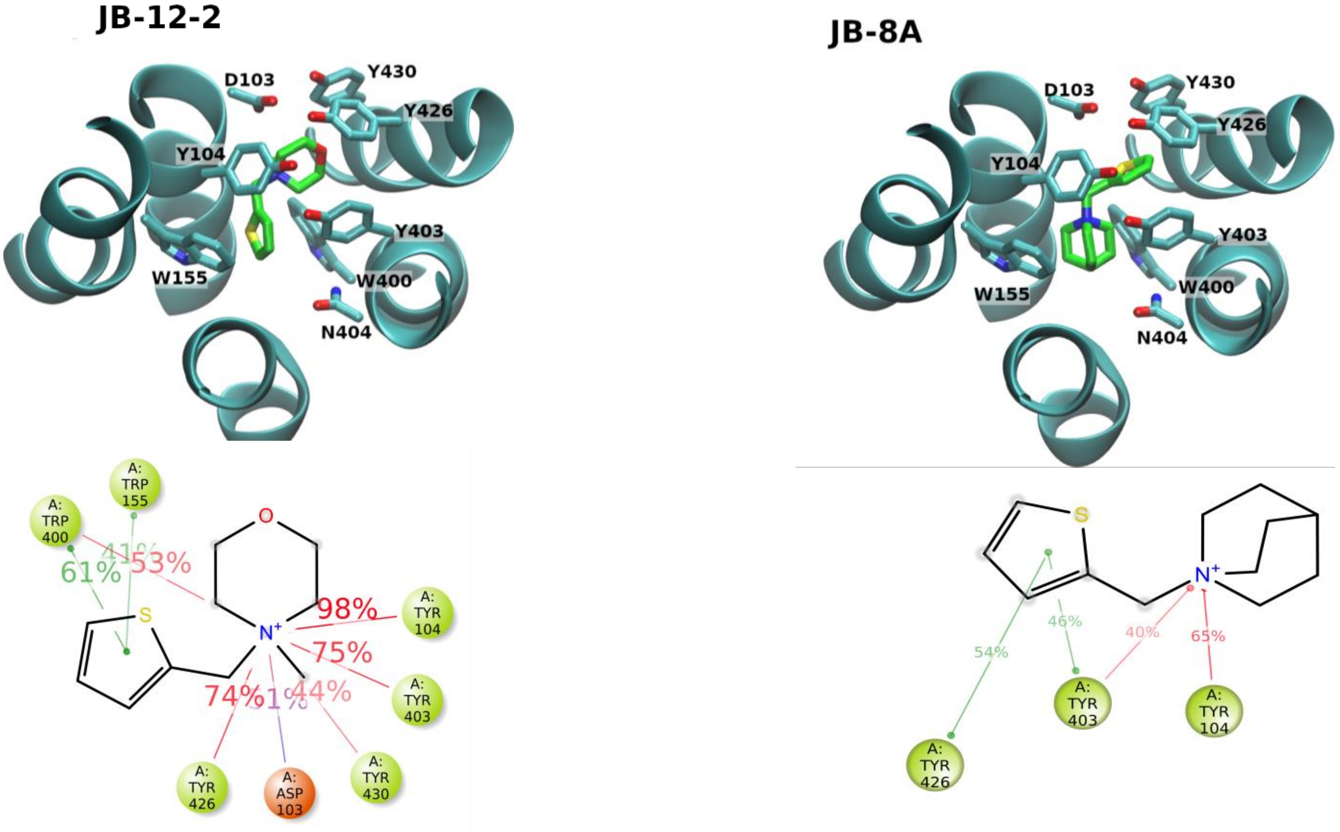

Figure S2B: Binding of JB-12-2 and JB-8A in the orthosteric binding site of M_2 receptor.

The model of the M_2 receptor in an active conformation (4MQS) was used. Top: The orientation of compounds JB-12-2 (left) and JB-8A (right) is shown. Bottom: Schematic of detailed JB-12-2 (left) and JB-8A (right) interactions with the protein residues. Interactions that occur more than 30% of the simulation time of molecular dynamics trajectory are shown.

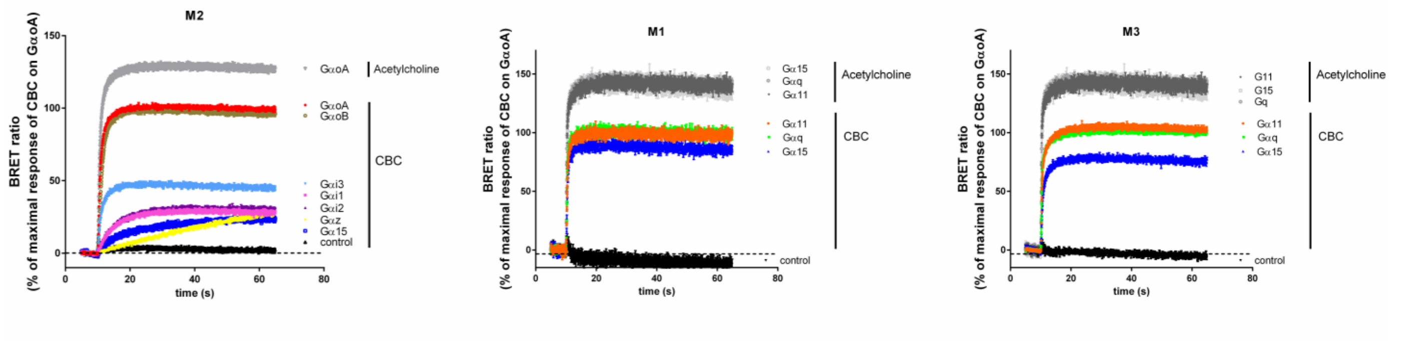

Fig. S3: Agonist-induced real-time monitoring of individual G-protein activation by the M2 (left), M1 (middle) and M3 (Right) receptors. Representative experiments performed in triplicates are shown. Responses to agonist CBC that were not transfected by individual Gα types are in black. Responses to agonist CBC at particular Gα (colour signal, as indicated in the picture legend) are shown. The grey signal is a response to the endogenous high-efficacy agonist acetylcholine. Values are mean ± S.E.M.

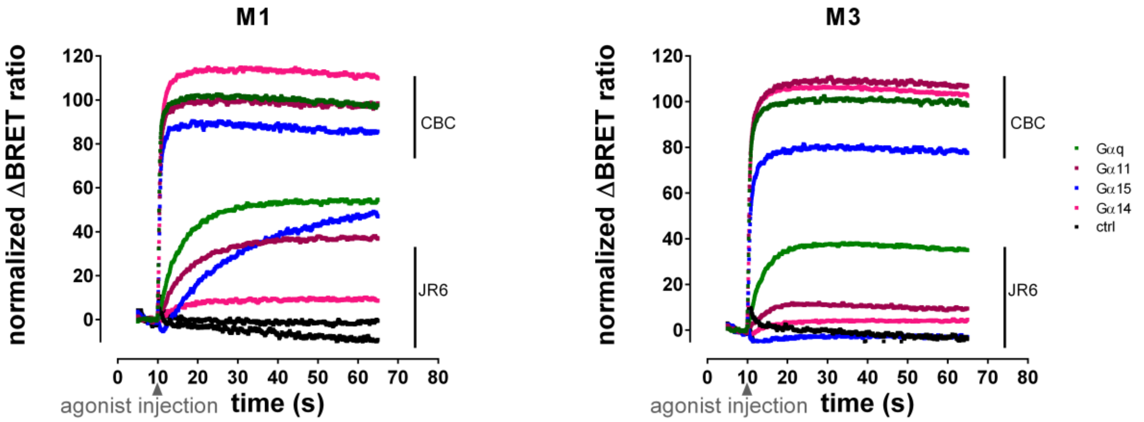

Fig. S4: Comparison of CBC and JR-6 induced activation of individual Gα via M1 and M3 receptors. Time-courses of the representative experiments of agonists-induced real-time monitoring of activation individual Gα subunits (indicated in the legend) induced by reference agonist CBC (100μM) and JR6 (100 μM) are plotted in the x,y graphs (X-axis, time in seconds; Y-axis, amplitudes of Gα activation expressed as ΔBRET ratio normalized to % of the maximal response of the reference agonist CBC at Gαq). Traces are running averages over 9 consecutive determinations.

Supplementary tables

Table S1: Parameters of agonists induced GTPγ[35S] binding to membranes from SF9 cells expressing M2 receptor and individual Gi/o Gα subunits. Parameters of functional response EC_{50} and E'_{MAX} were obtained by fitting Equation 3 to data from the measurement of GTPγ[35S] binding. Values of system E_{MAX} are (22.1 for M2+Gαi1; 23.02 for M2+Gαi2; 31.86 for M2+Gαi3; 17.95 for M2+GαoA; and 18.37 for M2_GαoB) Operational efficacy τ and agonist equilibrium dissociation constant K_A were calculated according to Equations 4 and 5 respectively. EC_{50} and K_A are expressed as logarithms. Values are means ± SD from three independent experiments performed in quadruplicates.

**Gi1**	EC50	E'max	Ka	tau
**CBC**	-4.78 ± 0.13	8.48 ± 2.42	-4.53 ± 0.04	0.63 ± 0.48
**Ixo**	-7.60 ± 0.06	11.79 ± 1.78	-7.26 ± 0.07	1.20 ± 0.43
**Oxo**	-5.76 ± 0.15	9.60 ± 1.51	-5.50 ± 0.10	0.79 ± 0.24
**Are**	-5.22 ± 0.10	5.36 ± 0.80	-5.06 ± 0.03	0.32 ± 0.07
**NDMC**	-6.33 ± 0.06	2.41 ± 0.35	-6.28 ± 0.05	0.12 ± 0.02
**McN**	-4.75 ± 0.09	2.11 ± 0.50	-4.71 ± 0.08	0.11 ± 0.03
**JR-6**	n.d.	n.r.	n.c.	n.c.
**PN-152**	n.d.	n.r.	n.c.	n.c.
**JB-8A**	n.d.	n.r.	n.c.	n.c.
**JB-12-2**	-3.50 ± 0.00	1.79 ± 0.01	-3.46 ± 0.00	0.09 ± 0.00
**JB-13-1**	-3.69 ± 0.16	1.58 ± 0.25	-3.66 ± 0.16	0.08 ± 0.01
**Gi2**	EC50	E'max	Ka	tau
**CBC**	-4.73 ± 0.15	10.33 ± 3.49	-4.46 ± 0.04	0.94 ± 0.54
**Ixo**	-7.48 ± 0.12	15.09 ± 3.80	-6.97 ± 0.12	2.49 ± 1.66
**Oxo**	-5.80 ± 0.23	14.37 ± 3.07	-5.32 ± 0.07	1.92 ± 0.99
**Are**	-5.13 ± 0.09	6.48 ± 2.68	-4.96 ± 0.05	0.42 ± 0.24
**NDMC**	-6.18 ± 0.15	3.05 ± 0.90	-6.12 ± 0.14	0.15 ± 0.05
**McN**	-4.82 ± 0.06	2.45 ± 0.68	-4.77 ± 0.05	0.12 ± 0.04
**JR-6**	n.d.	n.r.	n.c.	n.c.
**PN-152**	n.d.	n.r.	n.c.	n.c.
**JB-8A**	n.d.	n.r.	n.c.	n.c.
**JB-12-2**	-3.52 ± 0.04	2.30 ± 0.82	-3.48 ± 0.02	0.11 ± 0.04
**JB-13-1**	-3.79 ± 0.50	1.85 ± 0.85	-3.75 ± 0.48	0.09 ± 0.04
**Gi3**	EC50	E'max	Ka	tau
**CBC**	-4.86 ± 0.11	7.97 ± 4.79	-4.69 ± 0.05	0.39 ± 0.35
**Ixo**	-7.64 ± 0.10	17.22 ± 1.41	-7.27 ± 0.02	1.19 ± 0.21
**Oxo**	-5.88 ± 0.08	6.13 ± 0.95	-5.79 ± 0.07	0.24 ± 0.05
**Are**	-5.30 ± 0.04	6.96 ± 2.00	-5.19 ± 0.01	0.29 ± 0.10
**NDMC**	-6.17 ± 0.08	3.00 ± 0.79	-6.12 ± 0.07	0.10 ± 0.03
**McN**	-4.66 ± 0.15	2.47 ± 0.62	-4.62 ± 0.14	0.08 ± 0.02
**JR-6**	n.d.	n.r.	n.c.	n.c.
**PN-152**	n.d.	n.r.	n.c.	n.c.
**JB-8A**	n.d.	n.r.	n.c.	n.c.
**JB-12-2**	-3.61 ± 0.13	1.68 ± 0.13	-3.59 ± 0.13	0.06 ± 0.00
**JB-13-1**	-4.06 ± 0.21	1.42 ± 0.02	-4.04 ± 0.21	0.05 ± 0.00

GoA	EC50			E'max			Ka			tau		
CBC	-5.50	±	0.17	12.09	±	2.22	-4.99	±	0.03	2.52	±	1.41
Ixo	-8.15	±	0.09	14.20	±	1.81	-7.43	±	0.10	4.52	±	2.11
Oxo	-6.53	±	0.20	13.13	±	2.34	-5.93	±	0.07	3.29	±	1.59
Are	-5.80	±	0.18	10.61	±	1.43	-5.40	±	0.11	1.52	±	0.48
NDMC	-6.68	±	0.10	7.08	±	0.50	-6.46	±	0.08	0.65	±	0.08
McN	-5.04	±	0.17	5.84	±	1.51	-4.87	±	0.11	0.50	±	0.19
JR-6	-5.37	±	0.13	3.14	±	0.91	-5.28	±	0.10	0.22	±	0.08
PN-152	-5.79	±	0.42	2.70	±	0.18	-5.72	±	0.42	0.18	±	0.01
JB-8A	-5.45	±	0.24	2.04	±	0.34	-5.40	±	0.23	0.13	±	0.02
JB-12-2	-4.59	±	0.24	3.82	±	1.35	-4.48	±	0.20	0.28	±	0.12
JB-13-1	-4.23	±	0.10	4.34	±	0.28	-4.11	±	0.09	0.32	±	0.03

GoB	EC50			E'max			Ka			tau		
CBC	-5.20	±	0.15	11.93	±	2.64	-4.71	±	0.06	2.31	±	1.47
Ixo	-8.08	±	0.18	13.42	±	3.13	-7.46	±	0.09	3.72	±	2.48
Oxo	-6.26	±	0.21	12.43	±	4.52	-5.68	±	0.19	3.50	±	3.01
Are	-5.61	±	0.19	10.18	±	2.78	-5.24	±	0.06	1.40	±	0.69
NDMC	-6.44	±	0.16	5.43	±	0.69	-6.28	±	0.13	0.42	±	0.07
McN	-4.68	±	0.34	5.03	±	1.35	-4.54	±	0.31	0.39	±	0.14
JR-6	-5.38	±	0.11	2.89	±	0.41	-5.31	±	0.10	0.19	±	0.03
PN-152	-6.27	±	0.16	2.24	±	0.15	-6.22	±	0.16	0.14	±	0.01
JB-8A	-5.09	±	0.14	1.40	±	0.16	-5.06	±	0.13	0.08	±	0.01
JB-12-2	-3.62	±	0.07	3.13	±	0.50	-3.54	±	0.05	0.21	±	0.04
JB-13-1	-4.12	±	0.03	2.96	±	0.75	-4.04	±	0.01	0.19	±	0.06

Table S2: Relative intrinsic activities RAi of agonists at Gi/o Gα. RAi of tested agonists to reference agonist CBC were calculated, from parameters of functional response EC_{50} and E'_{MAX} (SI, Table S1) obtained by fitting Eq. 7 to data from measurement of GTPγ[35S] binding to membranes coexpressed M2 receptor with individual Gi/o Gα. Values of RAi are plotted in Fig. 4A.

	Gi1			Gi2			Gi3			GoA			GoB		
CBC		1			1			1			1			1	
Ixo	918.23	±	80.043	808.69	±	22.724	1320.6	±	59.009*	517.40	±	48.553	863.17	±	42.383
Oxo	10.747	±	0.478	16.427	±	1.507 *	8.179	±	0.257	11.543	±	0.950	11.917	±	0.073
Are	1.741	±	0.044	1.580	±	0.153	2.400	±	0.096	1.721	±	0.042	2.185	±	0.084
NDMC	10.119	±	0.202 *	8.217	±	0.394	7.658	±	0.517	8.789	±	0.627	7.882	±	0.148
McN	0.235	±	0.017	0.293	±	0.025 *	0.197	±	0.016	0.166	±	0.010	0.129	±	0.013
JR-6		n.c.			n.c.			n.c.		0.189	±	0.013	0.371	±	0.022 *
PN-152		n.c.			n.c.			n.c.		0.432	±	0.025	2.231	±	0.044 *
JB-8A		n.c.			n.c.			n.c.		0.149	±	0.001 *	0.091	±	0.005
JB-12-2	0.011	±	0.000	0.014	±	0.001	0.012	±	0.000	0.038	±	0.003 *	0.007	±	0.000
JB-13-1	0.015	±	0.001	0.020	±	0.000	0.029	±	0.002 *	0.019	±	0.000	0.021	±	0.000

n.c., not-calculated; *, greater than at other subtypes $P<0.05$, according to one-way ANOVA followed by Tukey´s multiple comparison test. Data are mean ± SD. Data from 3 independent experiments performed in quadruplicates were used in the calculation.

Table S3. Quantification of bias between two given Gi/o Gα. The bias factor was calculated, from parameters of functional response the equilibrium dissociation constant K_A and the operational efficacy τ (SI, Table S1) obtained by fitting Eq. 8 to data from measurement of GTPγ[^{35}S] binding to membranes coexpressing the M2 receptor and individual Gαi/o types. Values of bias factors between particular Gαi/o types are plotted in Fig. 4B.

control agonists					
Bias	**Iperoxo**	**Oxotremorine**	**Arecoline**	**NDMC**	**McN-A-343**
Gi2 vs Gi1	0.858 ± 0.258	1.269 ± 0.350	0.825 ± 0.220	0.710 ± 0.168	1.038 ± 0.253
GI3 vs Gi1	1.134 ± 0.315	0.654 ± 0.180	1.317 ± 0.370	0.667 ± 0.179	0.728 ± 0.206
GoA vs Gi1	0.495 ± 0.135	0.967 ± 0.260	0.904 ± 0.212	0.720 ± 0.147	0.597 ± 0.150
GoB vs Gi1	0.890 ± 0.274	1.211 ± 0.399	1.186 ± 0.316	0.638 ± 0.142	0.451 ± 0.119
Gi3 vs Gi2	1.322 ± 0.390	0.515 ± 0.144	1.596 ± 0.486	0.939 ± 0.255	0.701 ± 0.186
GoA vs Gi2	0.577 ± 0.167	0.762 ± 0.208	1.095 ± 0.283	1.013 ± 0.209	0.575 ± 0.134
GoB vs Gi2	1.037 ± 0.338	0.954 ± 0.317	1.438 ± 0.416	0.898 ± 0.202	0.435 ± 0.108
GoA vs Gi3	0.437 ± 0.117	1.479 ± 0.402	0.687 ± 0.187	1.079 ± 0.258	0.820 ± 0.223
GoB vs Gi3	0.785 ± 0.237	1.853 ± 0.615	0.901 ± 0.274	0.956 ± 0.247	0.620 ± 0.177
GoB vs GoA	1.797 ± 0.534	1.253 ± 0.408	1.312 ± 0.338	0.886 ± 0.171	0.756 ± 0.193
tetrahydropiridine-based agonists					
Bias	**JR-6**	**PN-152**	**JB-8A**	**JB-12-2**	**JB-13-1**
Gi2 vs Gi1	n.c.	n.c.	n.c.	1.061 ± 0.234	1.144 ± 0.315
GI3 vs Gi1	n.c.	n.c.	n.c.	0.947 ± 0.213	1.655 ± 0.404
GoA vs Gi1	inf.	inf.	inf.	2.923 ± 0.665	1.032 ± 0.213
GoB vs Gi1	inf.	inf.	inf.	0.504 ± 0.103	1.118 ± 0.270
Gi3 vs Gi2	n.c.	n.c.	n.c.	0.892 ± 0.226	1.447 ± 0.398
GoA vs Gi2	inf.	inf.	inf.	2.756 ± 0.704	0.902 ± 0.214
GoB vs Gi2	inf.	inf.	inf.	0.475 ± 0.110	0.977 ± 0.266
GoA vs Gi3	inf.	inf.	inf.	3.088 ± 0.802	0.623 ± 0.129
GoB vs Gi3	inf.	inf.	inf.	0.533 ± 0.125	0.675 ± 0.163
GoB vs GoA	1.913 ± 0.423	5.092 ± 0.935	0.603 ± 0.120	0.172 ± 0.041	1.083 ± 0.220

Data are mean ± SD from 3 independent experiments performed in quadruplicates. n.c., not-calculated; inf., infinity.

Table S4: Upper panel: Maximum amplitudes of Gαi/o and Gα15 activation at the M2 receptor. Data are expressed as % of maximum amplitude of reference agonists CBC on GαoA (CBC response at GαoA was set as 100%). Data are mean ± SD calculated from at least 3 independent experiments in triplicates. Lower panel: Activation rate. Data are expressed as activation rate constants K_{obs} in s^{-1}, calculated by one-phase association fit according to Eq. 9. from real-time monitoring of G-protein activation.

	maximal magnitude						
	GoA	GoB	G15	Gi1	Gi2	Gi3	Gz
CBC	100	89.09 ± 4.82 *	19.97 ± 4.28 *	24.20 ± 2.84 *	29.66 ± 1.55 *	41.48 ± 3.03 *	55.59 ± 5.89 *
JR6	13.95 ± 2.24	6.46 ± 1.93 #	n.r.	n.r.	n.r.	n.r.	n.r.
JB-12-2	11.81 ± 1.96	5.88 ± 1.05 #	n.r.	n.r.	n.r.	n.r.	n.r.
JB-13-1	10.77 ± 2.19	n.r.	n.r.	n.r.	n.r.	n.r.	n.r.
PN-152	m.r.	m.r.	n.r.	n.r.	n.r.	n.r.	n.r.
JB-8A	n.r.	n.r.	n.r.	n.r.	n.r.	n.r.	n.r.
	**activation rate Kobs [s-1]**						
	GoA	GoB	G15	Gi1	Gi2	Gi3	Gz
CBC	0.975 ± 0.093	0.660 ± 0.061#	0.094 ± 0.000#	0.208 ± 0.035#	0.183 ± 0.028#	0.714 ± 0.114#	0.012 ± 0.003#
JR6	0.078 ± 0.009	0.066 ± 0.025	n.r	n.r	n.r	n.r	n.r
JB-12-2	0.072 ± 0.003	0.066 ± 0.006	n.r	n.r	n.r	n.r	n.r
JB-13-1	0.074 ± 0.006	n.r	n.r	n.r	n.r	n.r	n.r
PN-152	m.r.	m.r.	n.r	n.r	n.r	n.r	n.r
JB-8A	n.r	n.r	n.r	n.r	n.r	n.r	n.r

n.r., no response; m.r., marginal response(less than 3% of CBC response). *, P<0.05 significantly different from the maximal response at GoA (set to 100) induced by CBC according to one-sample t-test; #, P<0.05 significantly different from the response of given agonist at GoA, according to one-way ANOVA followed by Dunnet´s multiple comparison test or t-test as appropriate.

Table S5: A) Maximum amplitudes of activation of individual Gαq/11 types. Maximum amplitude (ΔBRET ratio) was calculated by one-phase association fit according to Equation 7 from real-time monitoring of G-protein activation. Data are expressed as % of maximum amplitude of reference agonists CBC on Gαq. Data are mean ± SD calculated from at least 3 independent experiments in triplicates. B) Activation rates of individual Gαq/11 types. Data are expressed as activation rate constants K_{obs} in s^{-1}, calculated by one-phase association fit according to Eq. 9 from real-time monitoring of G-protein activation.

	maximal amplitude							
	Gαq		Gα11		Gα14		Gα15	
agonist	M1	M3	M1	M3	M1	M3	M1	M3
CBC	100	100	104.32 ± 3.65	103.60 ± 3.53	116.5 ± 5.63 *	101.52 ± 4.80	94.68 ± 7.68	76.68 ± 1.60 *
JR-6	59.72 ± 9.58	31.31 ± 9.68	43.25 ± 8.90	10.72 ± 6.38 #	10.75 ± 2.23 #	4.71 ± 0.72 #	47.09 ± 14.48	n.r.
JB-12-2	75.46 ± 12.62	45.27 ± 8.32	64.99 ± 8.88	20.83 ± 2.26 #	15.74 ± 0.85 #	10.61 ± 2.23 #	68.05 ± 13.11	20.63 ± 2.46 #
JB-13-1	n.r.	n.r.	n.r.	n.r.	n.r.	n.r.	n.r.	n.r.
PN-152	38.78 ± 6.74	10.61 ± 2.45	24.98 ± 4.77	n.r.	n.r.	n.r.	35.58 ± 8.52	n.r.
JB-8A	n.r.	n.r.	n.r.	n.r.	n.r.	n.r.	n.r.	n.r.
	**activation rate K obs [s-1]**							
	Gαq		Gα11		Gα14		Gα15	
agonist	M1	M3	M1	M3	M1	M3	M1	M3
CBC	2.21 ± 0.16	1.59 ± 0.21	1.90 ± 0.13 #	1.37 ± 0.17	1.26 ± 0.08 #	1.31 ± 0.11	1.70 ± 0.06 #	1.05 ± 0.09 #
JR6	0.17 ± 0.03	0.19 ± 0.06	0.15 ± 0.03	0.22 ± 0.08	0.16 ± 0.00	0.11 ± 0.01	0.04 ± 0.00 #	n.r.
JB-12-2	0.22 ± 0.02	0.23 ± 0.03	0.17 ± 0.02	0.24 ± 0.05	0.23 ± 0.07	0.23 ± 0.03	0.05 ± 0.00 #	0.03 ± 0.01 #
JB-13-1	n.r.	n.r.	n.r.	n.r.	n.r.	n.r.	n.r.	n.r.
PN-152	0.15 ± 0.01	0.20 ± 0.03	0.12 ± 0.02	n.r.	n.r.	n.r.	0.03 ± 0.02 #	n.r.
JB-8A	n.r.	n.r.	n.r.	n.r.	n.r.	n.r.	n.r.	n.r.

n.r., no response; * different from value100 (maximal response at Gq induced by CBC at given receptor) (P<0.05), according to one-sample t-test; # different from the response of a given agonist at Gq (P<0.05), according to one-way ANOVA followed by Tukey´s multiple comparison test

Table S6: Upper panel: Maximum amplitudes of non-preferential Gi/o Gα activation at M3 receptor. Data are expressed as % of maximum amplitude of reference agonists CBC on GαoA (CBC response at GoA was set as 100%). Data are mean ± SD calculated from 3 independent experiments in triplicates. Lower panel: Activation rates. Data from real-time monitoring of G-protein activation are expressed as activation rate constants K_{obs} in s^{-1}, calculated by one-phase association fit according to Eq. 9.

	maximal magnitude				
	**GoA**	**GoB**	**Gi1**	**Gi2**	**Gi3**
**CBC**	100	41.1 ± 8.384	16.56 ± 1.397	19.89 ± 1.673	54.38 ± 9.454
**JR6**	n.r.	n.r.	n.r.	n.r.	n.r.
**JB_12_2**	n.r.	n.r.	n.r.	n.r.	n.r.
**JB_13_1**	n.r.	n.r.	n.r.	n.r.	n.r.
**PN_152**	n.r.	n.r.	n.r.	n.r.	n.r.
**JB_8A**	n.r.	n.r.	n.r.	n.r.	n.r.
	**activation rate Kobs [s-1]**				
	**GoA**	**GoB**	**Gi1**	**Gi2**	**Gi3**
**CBC**	0.209 ± 0.006	0.081 ± 0.006	0.148 ± 0.001	0.12 ± 0.008	0.503 ± 0.046
**JR6**	n.r.	n.r.	n.r	n.r	n.r
**JB_12_2**	n.r.	n.r.	n.r	n.r	n.r
**JB_13_1**	n.r.	n.r.	n.r	n.r	n.r
**PN_152**	n.r.	n.r.	n.r	n.r	n.r
**JB_8A**	n.r.	n.r.	n.r	n.r	n.r

n.r., no response
